# China’s energy transitions for carbon neutrality: challenges and opportunities

**DOI:** 10.1007/s43979-022-00010-y

**Published:** 2022-04-19

**Authors:** Changying Zhao, Shenghong Ju, Yuan Xue, Tao Ren, Ya Ji, Xue Chen

**Affiliations:** China-UK Low Carbon College, Shanghai Jiao Tong University, Shanghai, 201306 China

**Keywords:** Carbon neutrality, China, Energy transition, Challenges, Opportunities

## Abstract

The pledge of achieving carbon peak before 2030 and carbon neutrality before 2060 is a strategic decision that responds to the inherent needs of China’s sustainable and high-quality development, and is an important driving force for promoting China’s ecological civilization constructions. As the consumption of fossil fuel energy is responsible for more than 90% of China’s greenhouse gases emissions, policies focusing on energy transition are vital for China accomplishing the goal of carbon neutrality. Considering the fact that China’s energy structure is dominated by fossil fuels, especially coal, it is urgent to accelerate the low-carbon transition of the energy system in a relatively short time, and dramatically increase the proportion of clean energy in the future energy supply. Although China has made notable progress in the clean energy transition in the past, its path to carbon neutrality still faces many significant challenges. During the process of energy transformation, advanced technologies and greater investment will play essential parts in this extensive and profound systemic reform for China’s economy and society. In the meantime, these changes will create immense economic opportunities and geopolitical advantages.

## Introduction

The Earth receives energy from the Sun in the form of short-wavelength electromagnetic radiation and simultaneously losses energy to outer space in the form of electromagnetic radiation as well but with much longer wavelengths. The Earth’s energy budget, describing the balance between the radiant energy that reaches the Earth from the Sun and the energy that flows from the Earth out to space, usually maintains a delicate and dynamic balance, and a small disturbance of this balance may cause significant consequences for the Earth’s climate system [1]. Commonly known as greenhouse gases (GHGs), certain atmospheric gases, such as carbon dioxide (CO_2_), water vapor (H_2_O) and methane (CH_4_), are critical to this energy balance. These GHGs are nearly transparent to the short-wavelength radiation, i.e. in the visible spectrum and ultraviolet radiation from the Sun, resulting in most of the solar radiation being absorbed by the Earth’s surface, i.e. land and oceans, while having a relatively high absorption rate for the infrared long-wavelength radiation emitted from the Earth, preventing some of the energy from escaping the Earth [2]. This so-called greenhouse effect has kept the Earth being warmer than it otherwise would be for billions of years, making it possible for life as we know it to evolve.

**Fig. 1:**
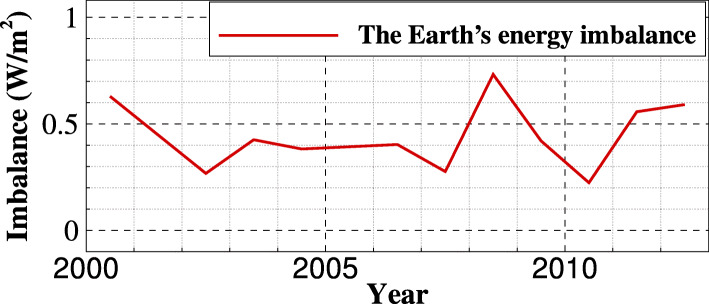
The global annual mean energy imbalances [5]

Climate observations and modellings have confirmed that the Earth is preserving more energy as heat from the Sun than it is radiating to space, even during the recent solar minimum [3–6]. Hansen et al. [3] reported that the inferred global annual mean energy imbalance is 0.58 ±0.15 W/m^2^ during the 6-year period 2005–2010. Stephens et al. [4] showed that the energy imbalance of the Earth for the approximate period 2000–2010 is 0.6 ±0.4 W/m^2^ on the top-of-atmosphere (TOA). The research group [5] further provided a summary of the energy imbalances over the past decade, in the form of the total global imbalance of the TOA flux, as presented in Fig. 1. These global energy imbalance researches have confirmed the positive trends in the total global energy imbalance. A recent NASA study [6] found that all-sky instantaneous radiative forcing has increased 0.53 ±0.11 W/m^2^ from 2003 to 2018, consistent with direct observations that anthropogenic activities are changing the Earth’s energy budget, trapping much more energy from the Sun than is escaping back.

**Fig. 2:**
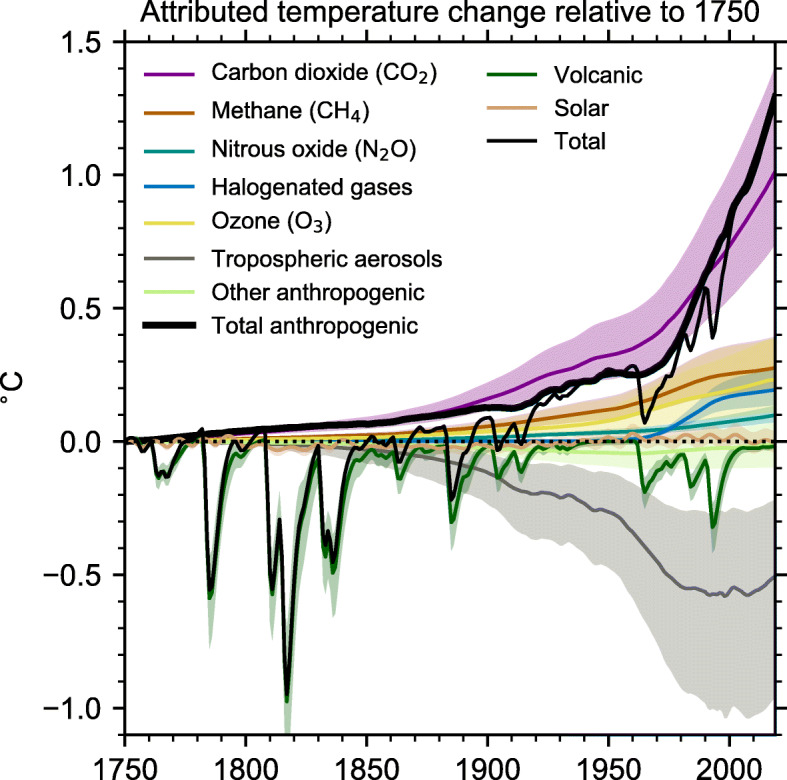
Attributed global near-surface air temperature change from 1750 to 2019 [7]

**Fig. 3:**
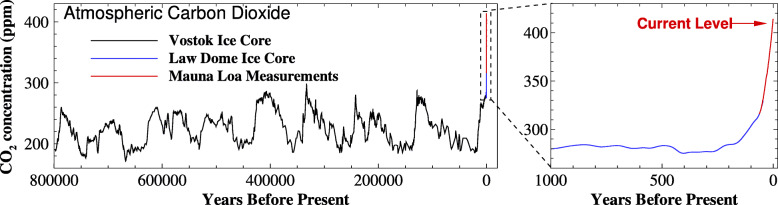
Global atmospheric carbon dioxide concentrations in parts per million (ppm) for the past 800,000 years (generated by the authors, data is from ftp.ncdc.noaa.gov)

In 2020, the global surface temperature was 1.19 ^∘^C higher than that of the pre-industrial period (1880-1900), which was the second-warmest year on record [8]. The Earth’s temperature has risen by 0.08 ^∘^C per decade since 1880, and the rate of warming over the past 40 years has been more than twice of that: 0.18 ^∘^C per decade since 1981 [8]. Recent global warming since the Industrial Revolution around 1750, as shown in Fig. 2, cannot be simply explained by natural causes, such as changes in solar activity, volcanic eruptions, and natural variations in GHGs concentrations, anthropogenic activities have contributed substantially to climate change by adding CO_2_, CH_4_, N_2_O and other halogenated gases such as hydrochlorofluorocarbons (HCFCs), to the atmosphere. As shown in Fig. 2, solar activity remains relatively stable over the last couple of centuries and volcanic eruptions only lead to temporary drops in global surface temperature. However, during the period of 1750 to 2020, atmospheric CO_2_ concentration is rising at an unprecedented rate. Figure 3 shows the global average CO_2_ concentration in the atmosphere over the past 800,000 years. For a very long period of time, CO_2_ concentration varies between roughly 180 and 300 ppm. However, over the last 100 years, CO_2_ concentration has dramatically increased and the number reached 412 ppm in 2020, which is approximately 47% above the level prior to Industrial Revolution and probably the highest at least in the past 800,000 years. The annual rate of increase in atmospheric CO_2_ over the past decade is also unprecedentedly high, which is about 2.4 ppm/year, according to the data released by the National Oceanic and Atmospheric Administration (NOAA) of the United States (https://gml.noaa.gov/). CO_2_ remains by far the most important positive anthropogenic global warming driving force, with CH_4_ the next most significant one. By 2020, the global annual mean concentrations of CH_4_ reached 1879.10 ppb, 159.6% increase since 1750 [9]. The global annual mean concentration of N_2_O has increased to a level of 333.03 ppb in 2020 [10]. The growth of N_2_O concentration since 1980 is largely attributed to a 30% increase in emissions from the expansion and intensification of global agriculture [7]. Some other trace gases, especially CFCs, have global warming potentials hundreds to thousands of times greater than CO_2_ and CH_4_, but are emitted in much smaller amounts. As a result, CO_2_ and CH_4_ remain by far the most important positive anthropogenic drivers, contributing 66.32% and 16.34%, respectively, of radiative forcing among all the GHGs in 2020 (https://gml.noaa.gov/dv/data.html).

In recent decades, the international community has begun paying close attention to global warming, aiming to tackle climate change and its negative impacts. On December 12^*th*^ 2015, world leaders at the United Nations Climate Change Conference (COP21) in Paris reached a breakthrough: the historic Paris Agreement [11]. The long-term goal of the Paris Agreement is to limit the rise in global average temperature to less than 2 ^∘^C above pre-industrial levels and pursue efforts to limit it to 1.5 ^∘^C. According to the latest reports from the Intergovernmental Panel on Climate Change (IPCC) [7, 12], 1.5 ^∘^C of warming is expected to occur by the mid-2030s and the world is on track for around 3 ^∘^C of warming by the end of the century without drastic change taken today for major reductions in GHGs emissions. To reduce GHG emissions, an increasing number of countries are making commitments to achieve carbon neutrality. Until November 10 ^*st*^ 2021, 137 countries have pledged to achieve carbon neutrality and 19 of the G20 members have set clear net-zero targets, as tracked by the Energy and Climate Intelligence Unit [13]. The governments constituting the G20, representing 80% of global gross domestic product (GDP), 60% of the global population, and are responsible for 77% of global GHG emissions, have a pivotal role to play in delivering stronger climate action and leading the transition that is required to limit temperature rise in line with the Paris Agreement [13, 14].

At the 75^*th*^ United Nations General Assembly on Sept. 22 ^*nd*^ 2020, China’s President Xi Jinping announced a concrete long-term target of peaking carbon emissions before 2030 and reaching carbon neutrality before 2060. Carbon neutrality is a major strategic decision made by China based on international and domestic overall interests. It is of great significance to China for constructing China’s ecological civilization, leading global climate governance, and achieving the “two 100-year” goals. Analysis [15] have shown that to achieve carbon neutrality before 2060, China needs to take immediate action, strive to follow the 1.5 ^∘^C pathway set in the Paris Agreement and work towards a 75% to 85% reduction in GHGs emissions by 2050. Considering the realities of China’s large total carbon emissions, high energy consumption demand, and “coal dominated” energy system, proposing a comprehensive carbon neutral plan and implementing courses of action, simultaneously meeting China’s social-economic development requirements, can effectively accelerate the process of China’s modernization. Primary consumption of fossil fuel energy is the leading source of more than 90% of China’s GHGs emissions, making energy policies a vital role in the country’s transition process to carbon neutrality. To realize carbon neutrality before 2060, China will have to dramatically boost its energy utilization efficiency, diversify the use of energy sources, improve operation efficiency, and implement carbon capture and storage technologies. Meanwhile, China will also have to increase public-private investment significantly to support its carbon neutrality agenda. Achieving carbon neutrality before 2060 is an indication of China’s great commitment to its energy transition. However, compared to developed countries, China as a developing country with a population of 1.4 billion, is facing greater challenges in achieving carbon neutrality before 2060, as energy demand will keep rising and the time from peak to zero emission is much shorter. The great challenges faced by China require strategic planning and urgent efforts, while also providing new opportunities for long-term and high-quality development through technological revolution and industrial upgrades.

## Carbon emission and climate policy overview

### Global carbon emission

**Fig. 4:**
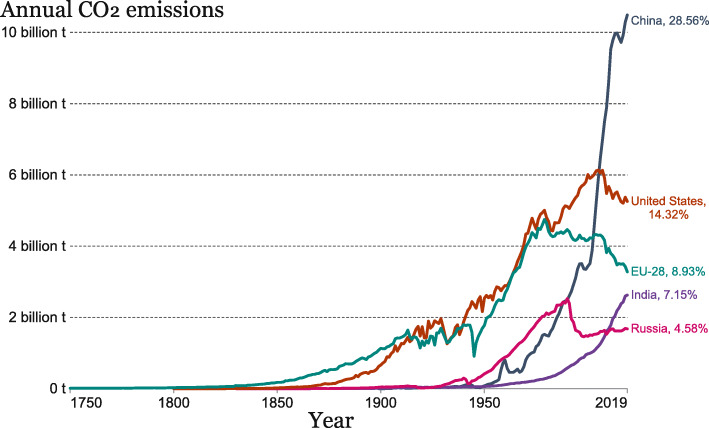
Major annual CO_2_ emitters between 1750 and 2019. (The figure is adapted from [16])

In 1950, the world emitted about 6 gigatonnes (Gt, billion tons) of CO_2_. By 1990, the value had almost quadrupled, reaching more than 22 Gt. And the global emission of CO_2_ further reached 36.44 Gt in 2019. Asia was the largest emitter, accounting for 56% (20.24 Gt) of global CO_2_ emissions. North America, dominated by the US, was the second largest regional emitter with 18% (6.48 Gt), which was followed closely by Europe with 15% (5.45 Gt) [16]. Counted on country basis, the global carbon emission was mostly contributed by China (28.56%), the US (14.32%), the EU-28 (European Union plus the UK, 8.93%), India (7.15%) and Russia (4.58%), as shown in Fig. 4. Although the growth has been slowing down over the last few years, the global CO_2_ emissions have not yet peaked. Based on the analysis of the change tendency and integrated factors of energy-related CO_2_ emission, International Energy Agency (IEA) projected that global CO_2_ emissions will grow by 4.8% in 2021, due to the rebounding demand for fossil fuels and the carbon-intensive economic recovery from the global financial crisis [17].

Considering the great global inequalities of carbon emissions from energy production and consumption, the exploration of CO_2_ emissions by country matters. Total annual, per-capita-based, and cumulative emissions by country often tell quite different stories, resulting in a confrontation over who can really make an influence: rich countries with high per-capita emissions, or those with a large population.

**Fig. 5:**
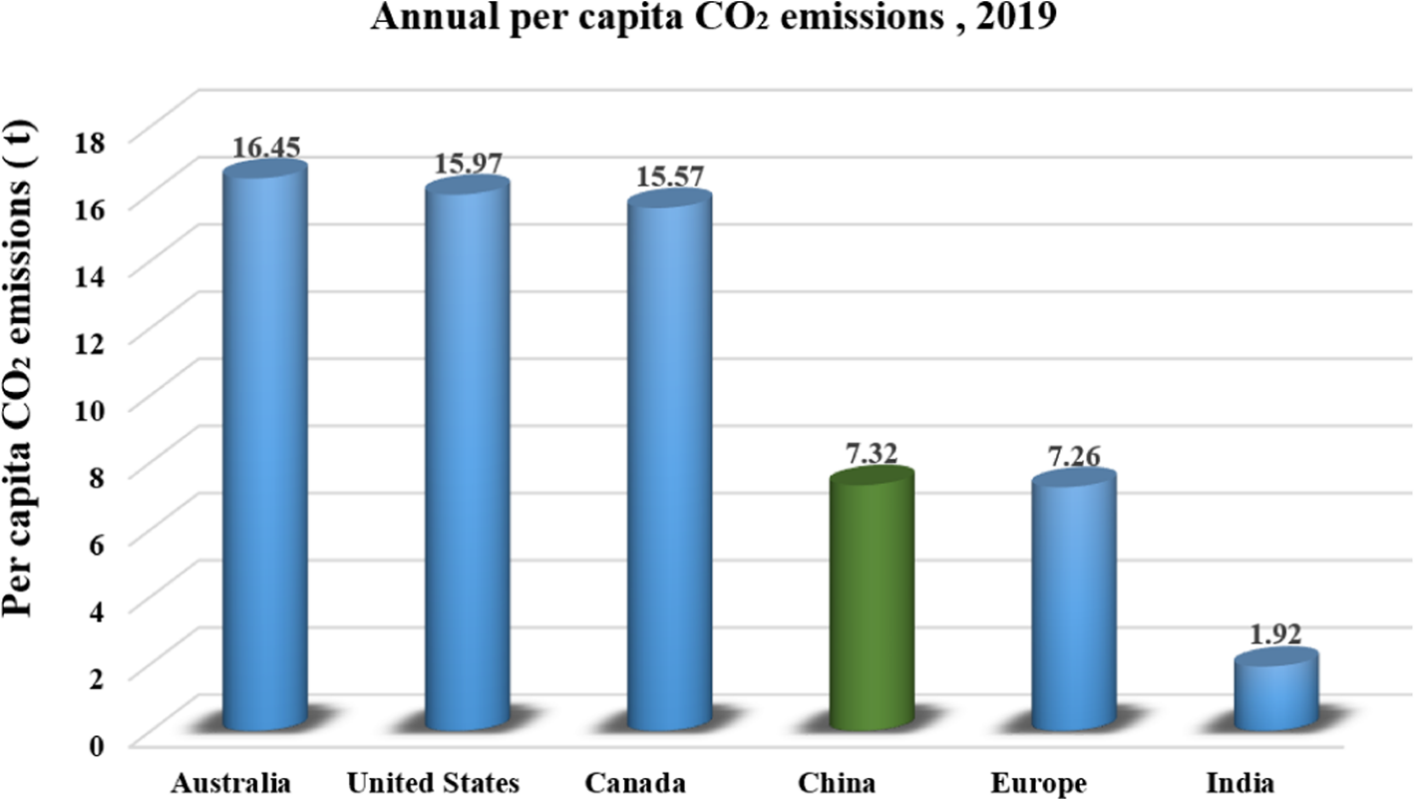
per-capita CO_2_ emission in 2019 (generated by the authors, data is from [16])

**Fig. 6:**
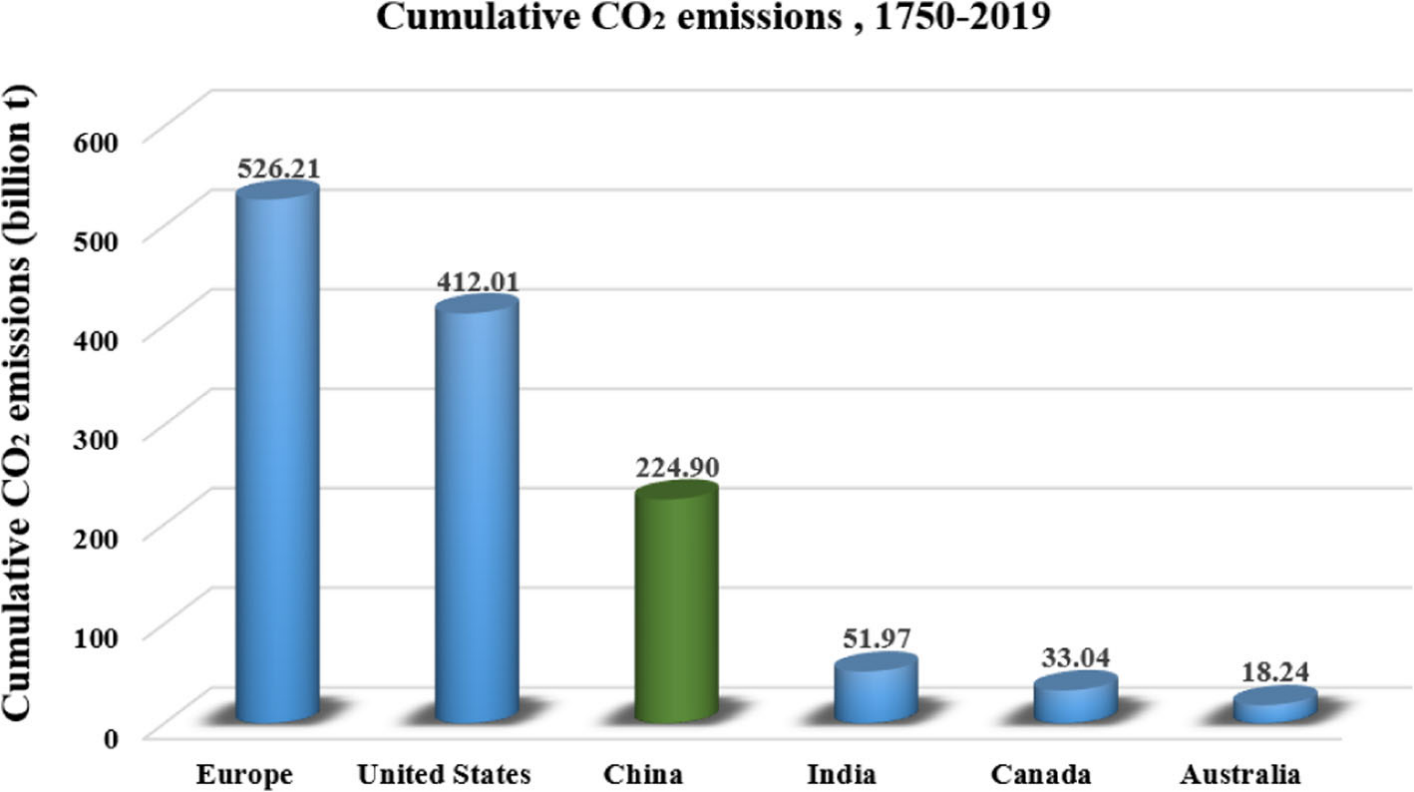
Cumulative CO_2_ emission during 1750-2019 (generated by the authors, data is from [16])

**Fig. 7:**
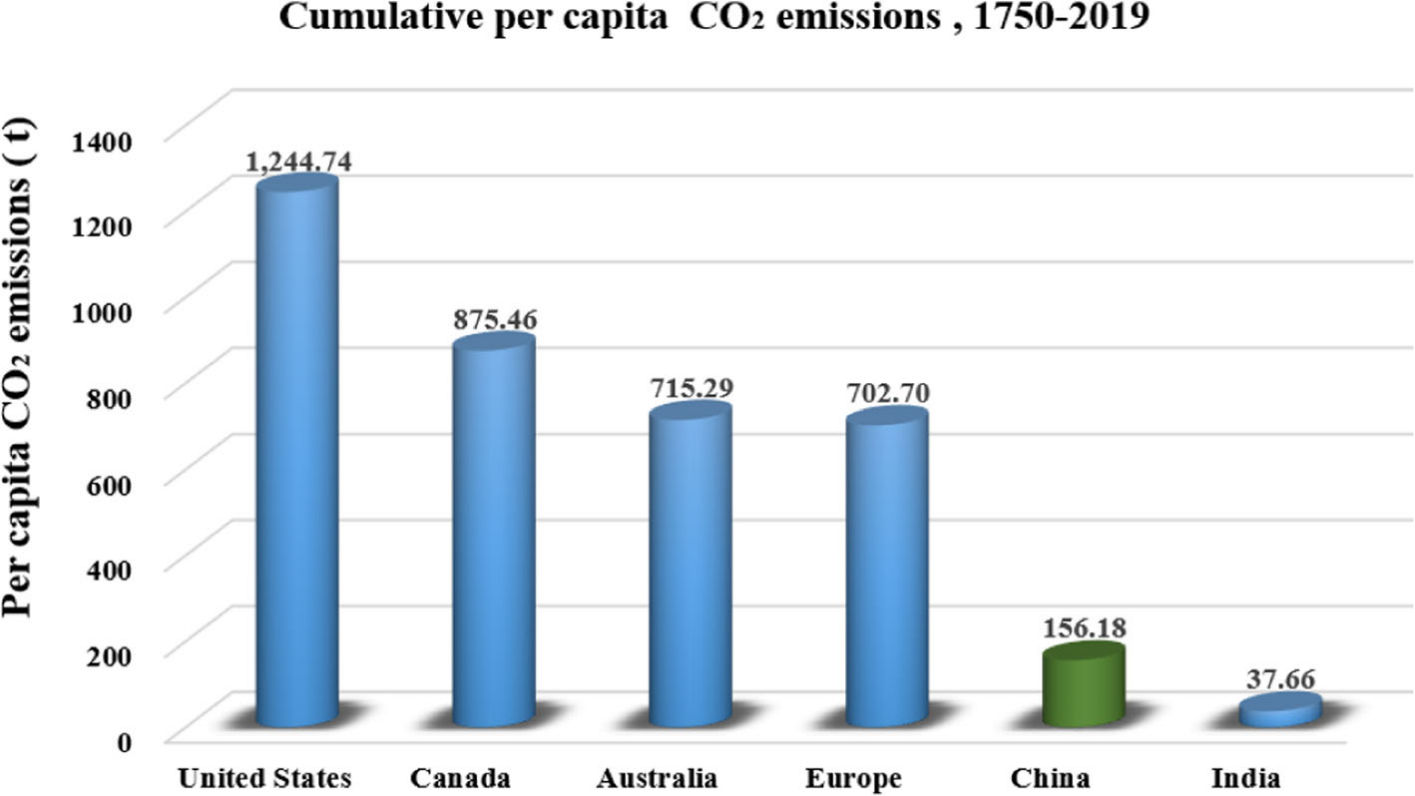
Cumulative per-capita CO_2_ emission during 1750-2019 (generated by the authors, data is from [16])

Figures 5, 6 and 7 illustrate per-capita emission, cumulative emission, and per-capita cumulative emission by different countries or regions. As depicted in Fig. 5, the United States, Australia, and Canada have relatively high per-capita emissions as well as large populations. Specifically, Australia has the highest per-capita carbon footprint of 16.45 tons, followed by the US with 15.97 tons, and Canada with 15.57 tons in 2019. These numbers are more than 3 times higher than the global average level of 4.72 tons. Generally, developed countries with better living standards would have higher carbon footprints, but the per-capita emissions can vary a lot even among these developed countries. For example, many European countries have much lower per-capita emissions than the US or Australia. Although the largest emitter in the world, Asia has slightly lower emissions per-capita compared to the global average. Similarly, China’s emission is the most significant margin in 2019, slightly higher than 10 Gt, the relating per-capita emission is much lower than that of the US, Australia and Canada. Apart from per-capita emission, the cumulative emission by country also reflects the great inequalities of carbon emission across the world. As shown in Fig. 6, developed countries, such as the US and the European Union countries, possess much higher cumulative carbon emissions than other countries. Allen et al. [18] have pointed out that limiting cumulative emissions of CO_2_ rather than emission rate or concentration level is more robust to scientific uncertainty about climate change. Thus, these developed rather than developing countries apparently should undertake more responsibilities in addressing global climate change. Additionally, information on the per-capita based cumulative carbon emissions (Fig. 7) further indicates the urgency of the implementation of a low carbon lifestyle for people in developed countries.

**Fig. 8:**
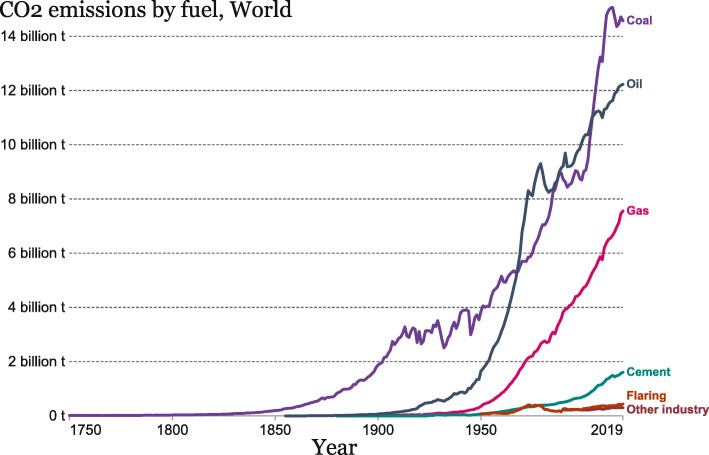
CO_2_ emission by fuel worldwide (the figure is adapted from [16])

The absolute contribution of CO_2_ emissions by sources over time is shown in Fig. 8. Early industrialization was supported by the combustion of solid fuel. Until the late 1800s, emissions from oil started to dominate the global CO_2_ emission. Recently, due to developing countries’ rapid industrialization process, coal rebounds to the top again, followed by oil and gas. And the US, EU-28, and Russia are currently the major contributors to the GHG emissions from oil and gas.

### China’s carbon emission

China’s energy supply heavily relies on coal, which contributes to the dramatic increase in carbon emissions over the last 20 years. In 2019, the carbon dioxide emission from coal was 7,888 Mt, 5.5 times that from oil (1,429 Mt), and 13.6 times that from natural gas (577 Mt). At the same time, the capacity of the coal power plants reached 1.04 billion kW, accounting for 50% of the global capacity. Power (including heat and electricity supply), industrial and transportation sectors contributed 47%, 28%, and 10% of domestic emissions, respectively. The emission shares of domestic power and industrial sectors in China are higher than the global average levels.

**Fig. 9:**
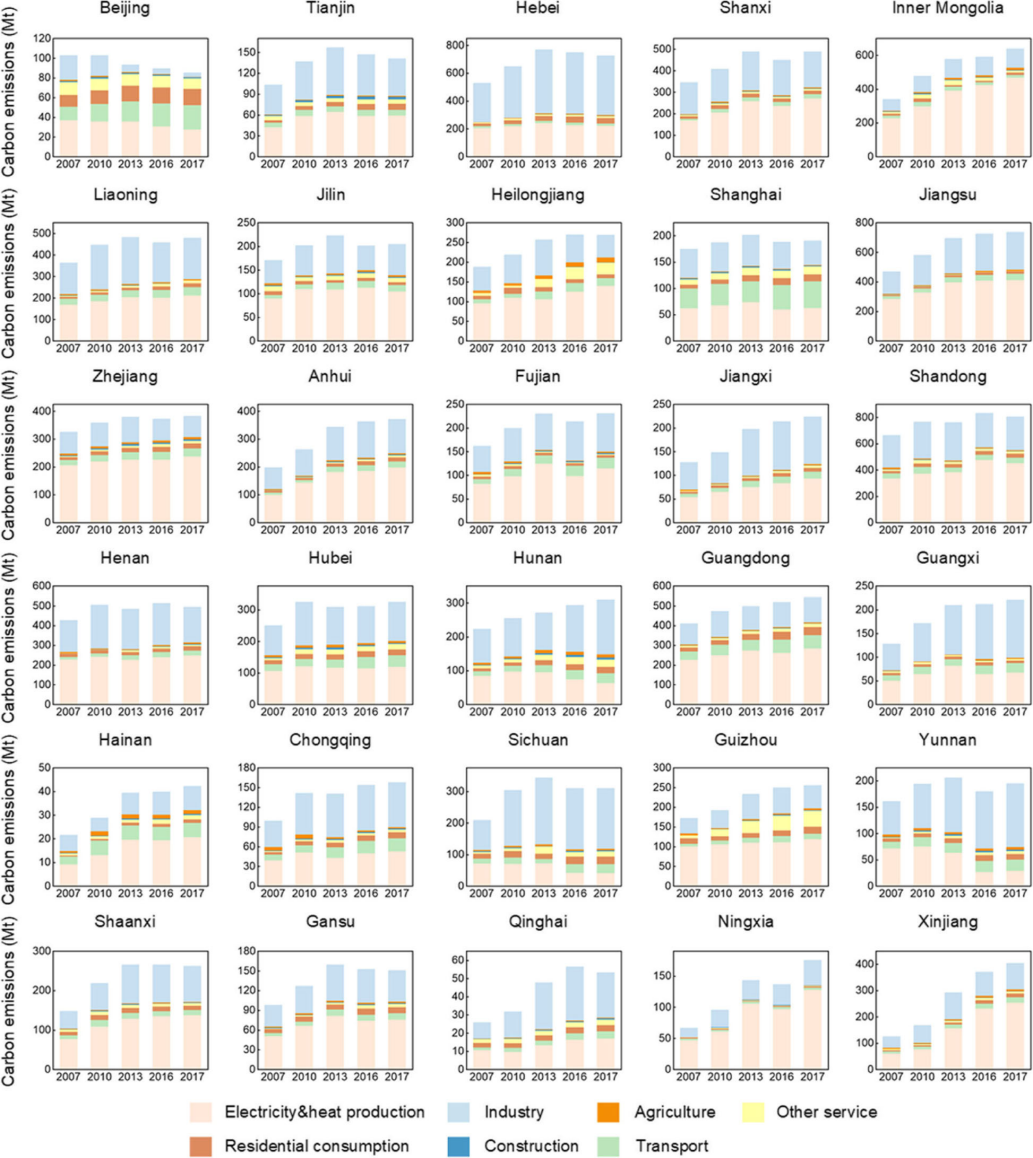
Sectoral carbon emissions in the 30 provinces from 2007 to 2017 [19]

Additionally, carbon emission in China also exhibits significant provincial differences (Table 1), in terms of the total amount, sectoral source, and changing trends. According to the results recently published by Mi and Sun [19], which is shown in Fig. 9, carbon emissions of provinces and cities like Beijing, Shanghai, Jiangsu and Tianjin, have already plateaued, indicating their readiness for achieving carbon peak in the relatively short term. And provinces characterized by rich-resource endowment, including Ningxia, Inner Mongolia, Xinjiang and Shanxi, are still experiencing dramatic increases in carbon emissions. As the degree of energy self-sufficiency is mostly above 1, it indicates that these provinces are undertaking carbon emission transfer from other provinces.

**Table 1:** Carbon intensity classified by provinces [21]

Classification	Province
Light emission province (12)	Beijing, Shanghai, Guangdong, Fujian, Zhejiang, Tianjin, Chongqing, Sichuan, Jiangsu, Hunan, Hubei, Hainan
Medium emission province(10)	Henan, Shandong, Jiangxi, Yunnan, Guangxi, Shaanxi, Jilin, Anhui, Heilongjiang, Guizhou
Heavy emission province(8)	Qinghai, Gansu, Liaoning, Hebei, Shanxi, Xinjiang, Inner Mongolia, Ningxia

Similar to Mi and Sun’s findings, Zhao’s research [20] also suggested that the developed region on the east coast of China has already reached the peak of fossil energy consumption. Investment in fixed assets contributed a big part to carbon emissions in this area. In the central region, the consumption of fossil fuels increased with economic development. And the emission of the western region is mostly contributed by heavy industrial enterprises. At the same time, due to the low level of economic development, the energy technology of industrial enterprises is relatively backward in the western region. Above all, different regions should combine their own development characteristics and location advantages, transform the development mode of energy-intensive economic growth, and continuously increase the share of renewable energy in future energy structures.

### China’s climate policy

**Fig. 10:**
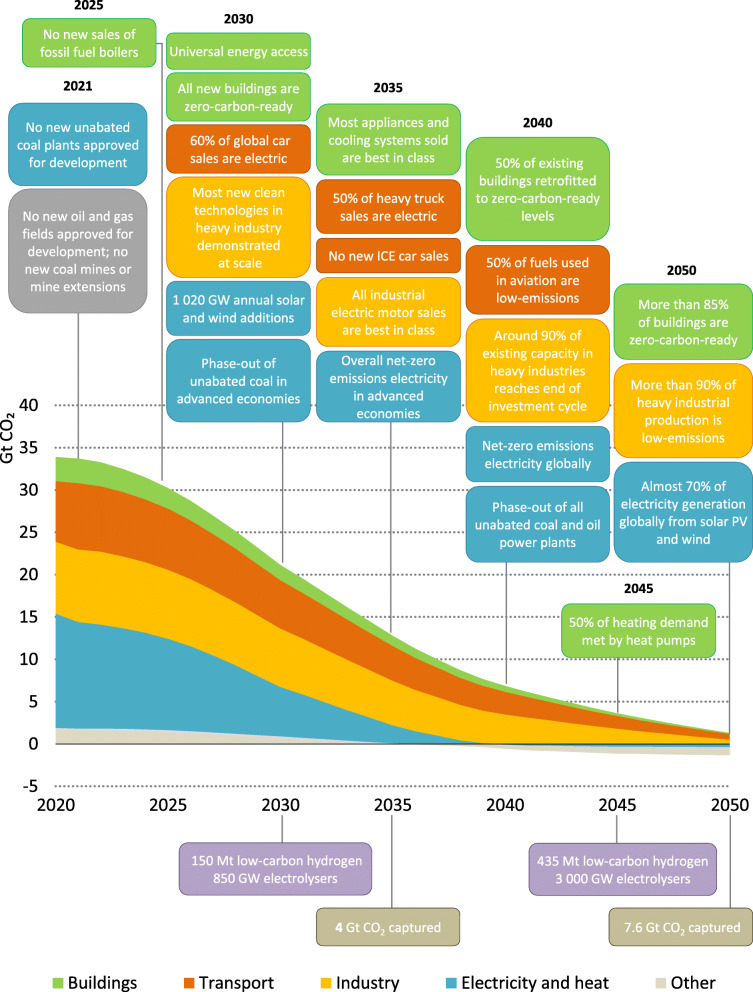
Key milestones in the pathway to net-zero [22]

According to the 6^*th*^ assessment report published by IPCC [7]: “It is unequivocal that human influence has warmed the atmosphere, ocean and land.” Major countries have proactively taken actions to address climate change. Up till now, more than 70% of the global economy has pledged carbon neutrality or net-zero emissions. As the energy sector is the main source of GHGs, IEA has proposed the net-zero pathway globally from 2020 to 2050, shown in Fig. 10. In the short term (2020-2030), the phasing-out of coal as the energy source should be the main focus of policymakers, while policies such as the adoption of renewable energy and the electric vehicle will gradually become the support for net-zero targets by the mid of this century. Although the commitments made by major countries sound solid, the dilemma between economic growth and carbon reduction still exerts many uncertainties on the accomplishment of long-term climate goals.

Unlike America’s capricious opinion about climate change and great uncertainty of climate policy, China consistently attaches great importance to climate issues. Recently, China’s central government announced two important documents “Working guidance for carbon dioxide peaking and carbon neutrality in full and faithful implementations of the new development philosophy” (Table 2) and “The action plan for carbon dioxide peaking before 2030” [23], signifying the determination of the whole country to combat climate change. The former defines the basic principles and main objectives in the future climate practice and will function as the top-level design for the low-carbon transition work. And the latter focuses on the following efforts in achieving high-quality carbon peaking:

- the adoption of innovation in green and low-carbon science and technology;

**Table 2:** Main objectives in the working guidance

Objective type	2025	2030	2060
Energy Intensity	13.5% decrease from 2020	–	–
Carbon Intensity	18% decrease from 2020	>65% decrease from 2005	–
Non-fossil share	20%	25%	80%
Carbon sink	Forest coverage 24.1%, total stock volume 18 billion m^3^	Forest coverage 25%, total stock volume 19 billion m^3^	–
Energy efficiency	Significant improvements in major industries	Major energy-intensive industries reaching international leading level	Domestic energy efficiencies reaching international leading level
- institutional reform in energy and other relevant fields;
- the development of incentives and constraint mechanisms;
- the assurance of maintaining energy security and economic development.

In addition to the aforementioned domestic efforts, the action plan also highlights international co-operations through global climate governance, green cooperation in business, technology, and finance, and making Belt and Road Initiative greener. Overall, these two documents mark the formation and continuous improvement of the “1+N” climate policy system in China. Currently, the policy package consists of several subsets, such as mitigation, adaption, synergistic management, financial policies, etc. During the last 10 years, the central government has given high priority to mitigation and adaptation policies which are summarized in the 2020 report published by the Ministry of Ecological Environment this year. Details are briefly introduced as follows:

- Adjust the industrial structure: reduce excessive capacity, and develop service and strategic-emerging industries;
- Save energy and promote energy efficiency: advance energy-saving in industrial, building and transportation sectors, and popularize energy-saving technology and product;
- Optimize energy structure: dual-control of energy consumption and energy intensity, advance clean use of fossil fuels and intensify the development of non-fossil fuel;
- Control emissions of non-CO_2_ GHGs in agricultural, industrial and waste management sectors;
- Increase carbon sinks in different eco-systems, including but not limited to the forest, grassland, wetland, agricultural land;
- Synergistic control of GHGs emissions and air pollutants emissions;
- Encourage regional low-carbon demonstrations.

By the end of 2019, China has decreased carbon intensity by 47.9% from 2005 and increased the share of nonfossil fuel to 15.3%, accomplishing the previously committed goals. For comparison, the US, Japan, and European Union only achieved 12%, 21% and 35% reduction in carbon intensity respectively. Guided by the 2030 carbon peak and 2060 carbon neutrality targets, China further expanded nationally determined contributions (NDC) to key commitments such as reducing 65% carbon intensity below 2005 levels by 2030 and increasing the forest stock volume by 6 billion cubic meters from the 2005 level.

Table 3 presents China’s key climate targets in the Five Year Plan (FYP) and NDC. As the programmatic document for China’s development in the next five years, the 14^*th*^ FYP sets the following climate-related targets by 2025 as reducing carbon and energy intensity by 18% and 13.5% from 2020 levels, increasing forest coverage to 24.1%, and increasing share of non-fossil sources in the energy mix to around 20%. The energy cap would be later published in the Special of 14^*th*^ FYP for Energy Development. Other related languages in 14^*th*^ FYP also indicate national determinations in addressing climate issues within the following fields: 1) controlling total energy consumption; 2) suppressing energy-intensive and high-emission projects; 3) promoting the development of hydropower and renewable energy; 4) developing new energy and intelligent vehicles; 5) establishing nation industrial innovation centers in strategic fields.

**Table 3:** China’s key climate targets in FYP and NDC

Target type	2009 NDC	2015 NDC	2021 NDC	13^*th*^ FYP (2016-2020)	14^*th*^ FYP (2021-2025)
Carbon Intensity	40-45% (2020) decrease from 2005	60-65% (2030) decrease from 2005	>65% (2030) decrease from 2005	18% decrease from 2015	18% decrease from 2020
Energy Intensity	–	–	–	15% decrease from 2015	13.5% decrease from 2020
Non-fossil share	–	20% by 2030	25% by 2030	15%	20%
Wind&Solar Capacity	–	–	1200 GW	–	–
Increased Forest stock volume from 2005	–	4.5 billion m^3^ by 2030	6 billion m^3^ by 2030	–	–

Among the ministries and commissions under the state council, the Ministry of Ecological and Environment (MEE) and the National Development and Reform Commission (NDRC) are highly active in launching climate policies in the aspects of emission control and energy consumption. On July 27^*th*^ 2021, MEE published the “Program for Starting Environmental Impact Assessment of Carbon Emission for Projects in Major Industries” [24]. In the program, MEE asked the local government to thrash out the methodology of carbon accounting for projects in major industries. Provinces including Hebei, Jilin, Zhejiang, Shandong, Shaanxi are included. And the assessment will initially start in industries including power, steel, architecture material, non-ferrous, petrochemical and chemical which are featured energy-intensive and highly polluting properties. As pointed out by Qian and Wu et al. [25], CO_2_ emission can be simultaneously mitigated under the framework of air pollution reduction policies. The implementation of the current program aims to strengthen the synergies between pollution control and carbon emission reduction.

Earlier this year, several provinces, such as Qinghai, Ningxia, Guangxi, Guangdong, Fujian, Yunnan, Jiangsu and Hubei were added to a list of highest level warning areas in terms of energy consumption. On September 11^*th*^ 2021, China’s National Development and Reform Commission issued the “dual control system” of total energy consumption and energy intensity [26]. The directive aims to perfect the current dual control system, improve the utilization efficiency and finally achieve the goal of decreasing the energy consumption intensity and optimizing the energy structure. The plan further proposed five actions in managing the total amount of energy consumption: 1) implement energy-consumption overall planning for key national projects; 2) resolutely manage and control energy-intensive and high-emission projects; 3) encourage local governments to increase consumption of renewable energy; 4) allow regional governments to be exempt from “dual control” assessments if regional governments hit a more strict energy intensity target (energy intensity is the priority); 5) promote the market-based trading system of energy-consumption indicators.

As coal is the key pillar in China’s energy structure but the major source of CO_2_ emission, proper management of coal consumption is vital for China to simultaneously secure its energy supply and reduce GHGs emissions. At the Leaders Climate Summit in April 2021, President Xi announced that coal consumption will be strictly controlled over the next 14^*th*^ Five-Year Plan (FYP) period and gradually phased down over the 15^*th*^ FYP. According to the China National Coal Association, coal consumption will stabilize at 4.2 Gt by the end of 14^*th*^ FYP. In September 2021, President Xi made the announcement at the United Nations General Assembly that China will not build new coal-fire projects abroad which previously ensure the electricity supply of developing countries under China’s Belt and Road Initiatives. Similar to China’s policies in controlling coal consumption, the US government also directed heads of agencies to identify fossil fuel subsidies and take steps to stop them, suspended oil and natural gas drilling leases in the Arctic National Wildlife Refuge, and revoked the permits for the Keystone XL pipeline. The US-China Joint Glasgow Declaration on Enhancing Climate Action in the 2020s issued on November 10^*th*^ 2021 further committed to tackling climate change with each country’s respective accelerated actions and cooperation in multilateral processes. Among EU countries, A coal phase-out is under discussion. Besides, a debate is ongoing that whether EU plants should invest more in pipes for gas imported from Russia and replace part of coal plants with gas plants. They also consider the significant potential to reduce natural gas demand, especially in the power and buildings sectors.

In the non-fossil energy supply, China’s updates NDC targets as increasing the share of non-fossil energy to 25%. As the leading country in developing solar and wind energy, the government also promised to enhance the total solar and wind capacity to 1200 GW by 2030. Meanwhile, the US government also required the domestic power providers to get a certain amount of their energy from fossil-fuel free sources and directed billion level support for renewables (mainly wind). Considering the limited space for wind and solar collection, the Japanese government emphasizes the change of energy mix by restarting/keeping a certain amount of nuclear (20-22%), increasing renewable generation, and promoting the usage of hydrogen and ammonia. As the pioneer region in addressing global climate change, the EU proposed a target of 40% of gross final consumption of energy being met from renewable sources by 2030, with an additional indicative share of 49% renewables in the building sector.

Additionally, two important policies published in 2020 are also important components of mitigation and adaptation climate policies. In June 2020, NDRC and the Ministry of Natural Resources unveiled a plan that includes major projects to protect and restore key ecosystems from 2021 to 2035. In efforts to protect biodiversity and enhance the ecosystem’s ability to fix carbon, 9 major projects and 47 key tasks have been underlined in the plan. In December 2020, MEE published “Measures for the Administration of Carbon Emission Trading (for Trial Implementation)” to instruct the construction of the national carbon emission market and regulate trading practices. Carbon trading together with “dual control system” are comprehensive policies in controlling carbon emission from both intensity and total emission basis.

Overall, emission control, energy consumption, carbon sequestration, and carbon trading will be four pillars supporting the implementation of China’s future climate policies. Currently, the prototype of the climate policy system has already come into effect and the energy transition is critically important in future practice.

## Energy transition pathways with profound revolution

### Status and challenges

Although China currently is the world’s largest CO_2_ emitter, the cumulative and per-capita-based emissions are far less than those of developed countries, resulted from a much shorter industrialization process of China. As shown in Fig. 6, the cumulative CO_2_ emissions between 1750-2019 from China is only about half of the values of the United States. As the world’s largest economy, the US has an average CO_2_ emission of 15.97 tons per person in 2019, more than twice that of China (Fig. 5).

**Fig. 11:**
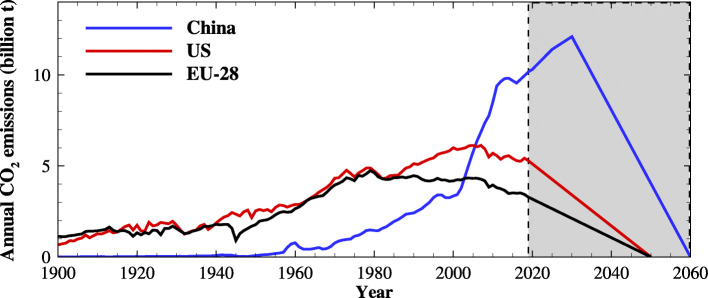
Annual CO_2_ emissions for China, the US and EU-28. Historical CO_2_ emission data before 2019 is from [27] and the projected data does not guarantee future results, which is only illustrative

Though with much lower cumulative and per-capita-based emissions, China still faces tremendous challenges in reducing carbon emissions, as the time remaining for the transition from its carbon peak toward neutrality will be much shorter than that of developed countries. As shown in Fig. 11, China’s carbon emission is currently about 10 Gt and has not reached its peak yet. And the EU-28 countries peaked the carbon emissions in the 1980s, reaching its net-zero target in 2050 from a much lower peak takes about 70 years. The time for the US to carbon neutrality from its peak at about 6 Gt in 2007 still lasts for more than 40 years. By comparing the 2019 emission level with 1990, Europe’s CO_2_ emissions have decreased by almost 20%; the US’s CO_2_ emissions declined from the 2007 peak to the same level as in 1990; while China’s CO_2_ emissions increased more than three times at the same time. If as have pledged that achieve carbon peak before 2030, China will only have about 30 years to achieve carbon neutrality after reaching a much higher peak than those of the US and EU. The longer China’s primary energy consumption and CO_2_ emissions continue to rise and the larger the peak values become, the more difficult it will be for China to achieve carbon neutrality in the future. In order to achieve the goal of carbon neutrality, China needs to make prompt adjustments to economic and social development and pay huge prices.

**Fig. 12:**
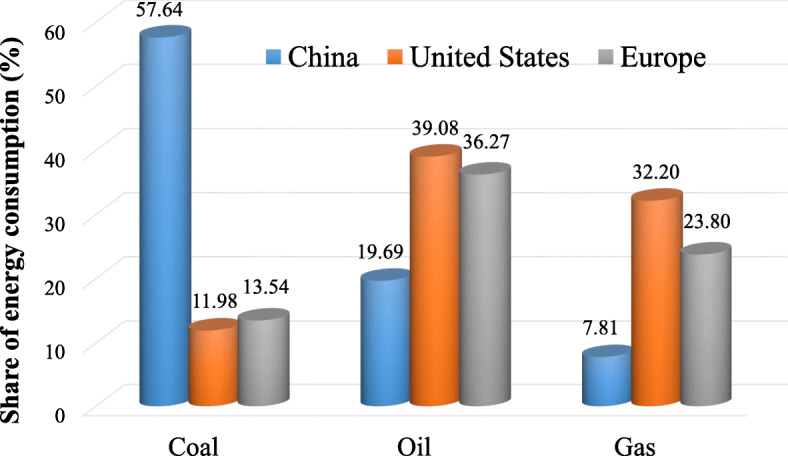
Share of the primary energy consumption by sources of China, United States and Europe in 2019 (generated by the authors, data is from [27])

In addition, China’s energy structure is still dominated by high-carbon energy and China’s economy is heavily dependent on coal. China’s energy supply is dominated by fossil energy, accounting for as high as 85% of the primary energy consumption and 92% of the total CO_2_ emissions, and the energy utilization efficiency is relatively low. As shown in Fig. 12, coal accounts for 57.64% of the total primary energy consumption in China in 2019, while Europe and the United States only consumed about 13.54% and 11.98% of coal in the respective primary energy mix. According to the IEA’s prediction [28], China will consume 3,875 Mt of coal in 2021, which accounts for more than 52% of global coal consumption.

**Fig. 13:**
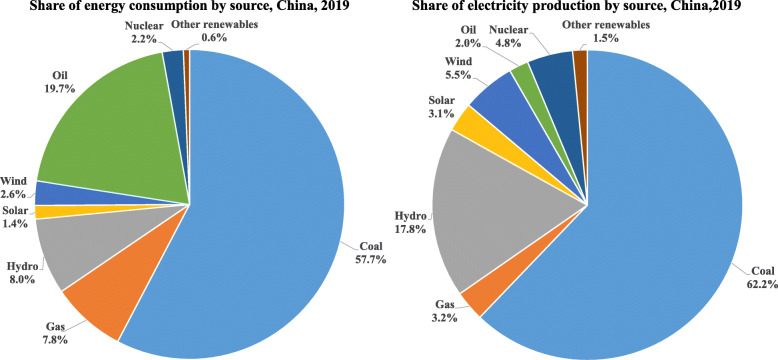
Share electricity production by source, China, 2019 (generated by the authors, data is from [27])

**Fig. 14:**
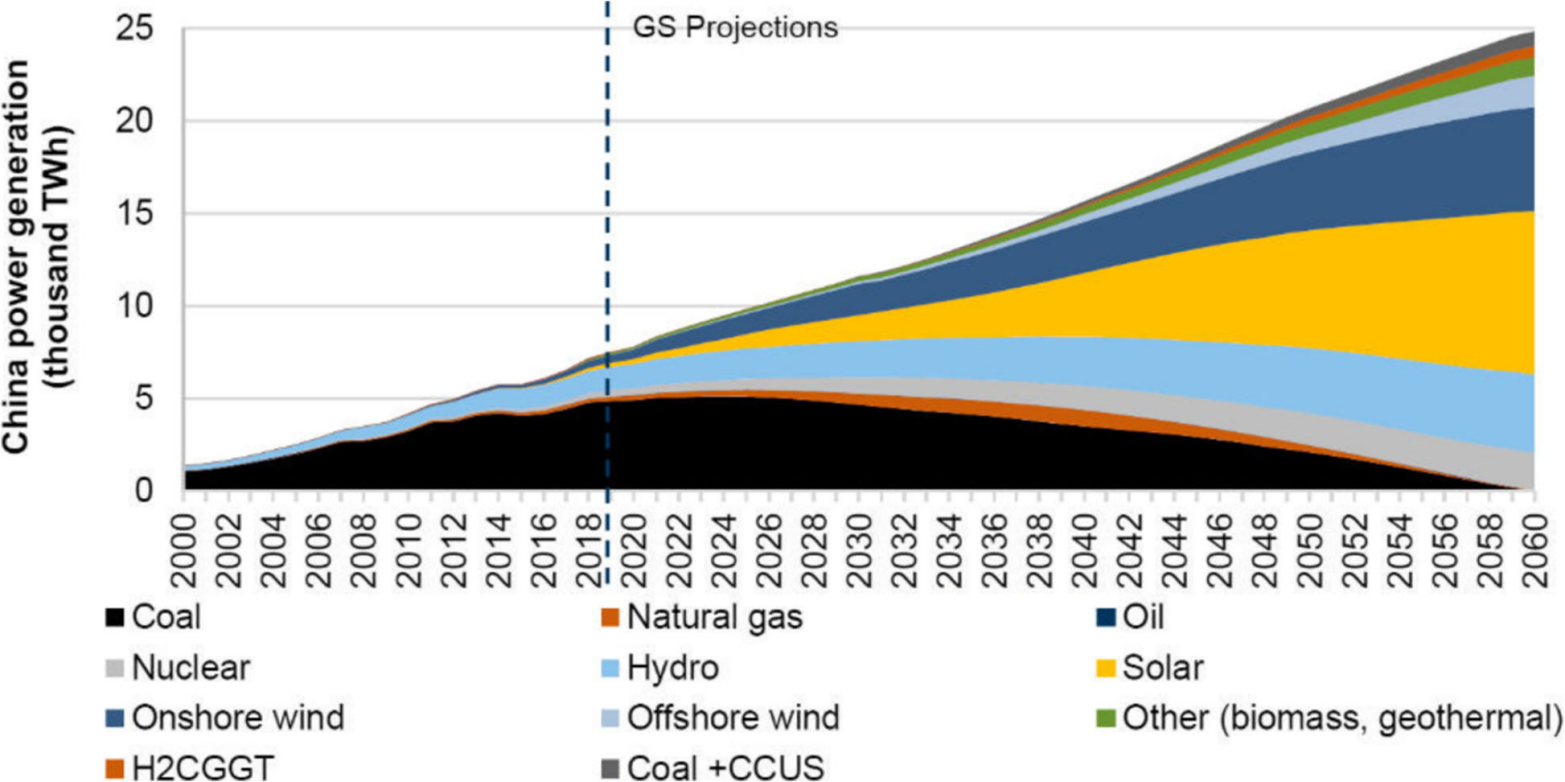
China’s electricity generation [37]

Figure 13 presents the share of primary energy consumption and share of electricity production by different sources of China in 2019. Fossil fuel-fired electricity generation accounts for about 67% of the total power generation, while coal-fired power generation alone takes more than 62%. On the contrary, only 17.5% Europe’s electricity production is from coal and that number is 23.2% for the US. In order to achieve carbon neutrality for China, coal-fired power generation is expected to be phased out by 2050. Coal has to be replaced by wind, solar, hydro, and nuclear energy. The share of fossil energy is expected to decline to less than 20% in 2060 (Fig. 14). Reducing dependencies on energy and electricity generation from high-carbon sources will be a major challenge in combating climate change because of its price advantages and the relatively young age of coal-fired power plants in China [15]. Furthermore, China is still in the industrialization process, the demand for energy and electricity will continue to rise and may plateau for a relatively long time. Currently, there are still strong coupling relationships among economic development, energy consumption, and carbon emissions in China.

The strong coupling of economic development and carbon emissions also means the transition from fossil fuel-based energy systems to renewables will also have significant socio-economic impacts. In a long run, it would enable faster growth of the economy, create more jobs, improve the overall welfare of a country, and has positive demographic and geopolitics impacts. But these socio-economic benefits require a rapid but smooth transition. Therefore, the realization of China’s carbon-neutral vision must be under the premise of sustained and stable economic growth, and a pragmatic path that can not only ensure the safe and reliable supply of energy and electricity but also achieve carbon emission reduction. This is a very serious challenge on the path of china’s carbon neutrality mission.

### Energy transition pathways

To achieve the goal of reaching a peak in CO_2_ emissions before 2030 and carbon neutrality before 2060, the energy system would be reshaped by the energy transition in China. The low-carbon transition of energy systems is imperative to achieve the goal. Carbon neutrality can be realized by reducing CO_2_ emissions directly by reducing consumption of traditional fossil fuels and offsetting CO_2_ emissions by negative emissions produced by bioenergy, in conjunction with carbon capture, utilization, and storage (CCUS), and direct air capture of CO_2_ with storage [29, 30]. There are a lot of pathways for energy transitions involving different rates of change, different aspects of the transformation of the energy system and many uncertainties. To achieve a peak in CO_2_ emissions as soon as possible, the government of China controls the peak quantity of fossil fuels [31]. Therefore, different pathways have the same trend that the share of low carbon energy such as solar, wind and other renewable sources jumps from 15% today to over 80% in 2060 [32].

According to the IEA’s announced pledges scenario (APS) [32], coal makes up 57% of the primary energy system in 2020 among various primary energy sources, bringing large amounts of CO_2_ emissions. Coal reduction in the energy transition is of great importance [33]. The ratio of fossil fuels in the total primary energy systems will decrease to less than 20% in 2060; they will be used mainly as supplemented energy sources in 2060 and the emissions will be removed by CCUS technology to achieve net-zero emissions [34]. Among different renewable energies, solar energy is one of the dominant energy sources. Solar energy is utilized for both power generation and heating by technologies of solar photovoltaic (PV) and solar thermal, accounting for a quarter of total primary energy demand in 2060 [35].

China’s total energy consumption from 2020 to 2060 is as important as primary energy demand to achieve peak in CO_2_ emissions before 2030 and net-zero CO_2_ emissions before 2060. Coal demand in final energy shows the biggest decline, falling almost 85% between 2020 and 2060 while electricity and hydrogen increase the most in the final energy system [32]. Electricity consumption demand is almost doubled in 2060 compared to 2020. Final energy demand mainly lies in power generation, industry, buildings and transport sectors. Electricity is the leading energy source in 2060. Electricity generation increases by 130% by 2060. In the industry sector such as cement and steel, coal is replaced by low-carbon fuels including bioenergy and waste and hydrogen. In the transport sector, oil demand falls 60% while hydrogen, ammonia and synthetic hydrocarbon fuels cover almost 25% of all energy needs in 2060 and electricity around 55% [32]. In the buildings sector, fossil fuels are mainly replaced by electricity and renewable energy. It is obvious that electrification is a critical component in main sectors to realize decarbonization and net-zero CO_2_ emissions. Electrification refers to the process of replacing technologies that use fossil fuels with technologies that use electricity as a source of energy [36]. Electrification is realized in different sectors such as factory electrification and household electrification. In the goal of carbon neutrality, electrification performs a significant and irreplaceable role. In China, the share of electricity in total final energy consumption is more than 50% in 2060. Electricity is highly demanded in sectors of industry, buildings, transportation and hydrogen generation.

**Fig. 15:**
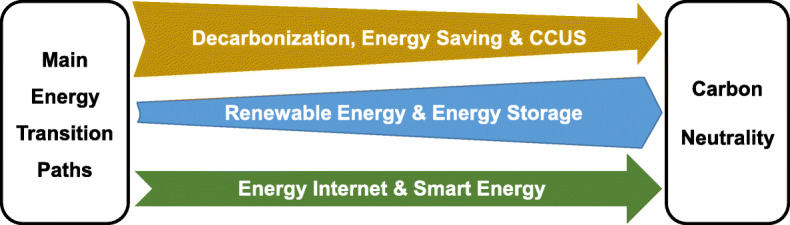
Roadmap of energy transitions for carbon neutrality in China

On the path of the energy transition for carbon neutrality, there are various types of emission reduction and low carbon technologies involved, including high-efficiency and low-emission combustion power generation technology, CCUS technology, high-efficiency catalytic conversion of carbon-based energy technology, and renewable energy utilization technologies such as hydrogen, solar, and wind energy. Figure 15 shows the three main energy transition paths for carbon neutrality in China. First, decarbonization, energy saving and CCUS are important energy transition ways to reduce carbon emission at an early stage since fossil energy accounts for 85% of the primary energy consumption. However, the CCUS technology is still immature and the cost is currently high, it is forecast to be widely used after 2035. With the increase of wind and solar power capacity, the combination of renewable sustainable energy and energy storage technology will become more and more important. The two energy transition paths will coexist for a long term, supporting and supplementing each other. At the same time, the energy internet and smart energy, which combines multiple renewable energy systems, data and information technology and carbon emission management, further contributes to the energy transition and emission reduction.

#### Different sectors: industry, transport and building

As described above, several key sectors contribute to most CO_2_ emissions so it is important to analyze energy transition pathways in key sectors including electricity generation, industry, transport and building sectors. Electricity generation has been discussed above. In this section, the industry, transport and building sectors are being reviewed. CO_2_ emissions in the industry account for 35% of China’s total energy sector emissions in 2020. There are several prominent changes in industrial CO_2_ emissions mainly from cement, steel and chemicals sectors between 2020 and 2060, showing a rapidly decline trend [38]. Industrial CO_2_ emissions decrease by 95% by 2060 due to electrification, energy efficiency improvements, hydrogen and CCUS [39] in industry. The decreasing trend of industrial CO_2_ emissions is consistent with the trend of final energy consumption in China with decreasing coal and increasing electricity consumption. Reducing coal used in industry results in a direct decrease in CO_2_ emissions while increasing electricity consumption results indirectly. Both two trends contribute to energy transition in the industry positively.

China has a great share in the global production of major bulk materials. Crude steel and cement produced by China account for more than half of global production in 2020 and both drop to 30% of the global share in 2060 during energy transition. In China, steel demand has increased rapidly over the past two decades due to infrastructure needs. In 2020, China’s steel production reaches a record of 1.1 Gt despite the Covid-19 pandemic. During steel production in China, coke and coal are used as reduction agents to cleave the oxygen from iron ore to produce molten iron. Except for the steel produced from carbon-based reducing agents, steel can be also produced by using electric furnaces which is typically used for scrap steel [40]. Scrap steel with low carbon emissions will grow rapidly in the coming decades. By 2060, electric furnaces play the main role in steel production, facilitating the steel sector’s transition to using electricity [41]. Besides scrap steel, hydrogen-based direct reduced iron is an important part in the steel sector, which uses hydrogen as a reducing agent substituting coal and coke [42]. This method offers advantages of high energy efficiency and low cost by using electricity from renewable energy. Crude steel production equipped with CCUS also contributes to energy transition in the steel sector, including innovative smelting reduction with CCUS and innovative blast furnace steelmaking with CCUS [43]. The abovementioned routes make up more than two-thirds of primary steel production and scrap-based electric arc furnace production is more than half of total steel production by 2060. China has the biggest share of cement in the global market. More than half of global cement is produced by China. The production of cement is responsible for one-third of China’s overall industrial CO_2_ emissions [44]. China’s cement production still grows in the near future due to demand for infrastructure and building and then begins to fall after peaking. Cement is produced from limestone in dry kilns with coal as energy input, thus emitting large amounts of CO_2_. By reducing the clinker-to-cement ratio, increasing material and energy efficiency and replacing coal with alternative fuels such as hydrogen and bioenergy blending, CO_2_ emissions decreases in 2030 and continues to decrease sharps with the help of CCUS [45]. More than 80% of China’s cement production is equipped with CCUS by 2060.

China’s transport sector emitted 9% of the total energy sector CO_2_ emissions in 2020 [46]. CO_2_ emissions from transport reach a peak in 2030 and then drop by 90% in 2060 compared to 2020. The peak in 2030 is predicted by spectacular growth in car ownership. The sharp decline of CO_2_ emissions is mainly from cars, buses and freight trucks, driven by policy efforts to adopt low-carbon technologies across various transport mode [47]. Decarbonization in all main transport modes in China is the key to achieving carbon neutrality. Gasoline and diesel used in most vehicles are substituted by electricity and hydrogen. Modal shifts from cars to public transport and non-motorized mode also play an important role in decarbonization.

Energy and carbon emissions in the building sector raise much attention nowadays. In China, buildings accounted for 20% of total CO_2_ emissions in 2020, resulting from providing heat and electricity for various buildings [48]. In the global view, China accounted for 17% of global energy consumption and 25% of CO_2_ emissions in the building sector in 2020. Residential properties including heating and cooling, cooking, electrical appliances and lighting are the sources of direct and indirect CO_2_ emissions in buildings [49]. The direct use of coal and traditional biomass has decreased rapidly in recent years. Electricity is the dominant energy in the building sector in 2060, propelling reduced emissions. Besides, renewable energy also promotes reduced CO_2_ emissions by employing solar PV in buildings, getting electricity from solar energy instead of public networks.

### Key technologies

#### Solar energy

**Fig. 16:**
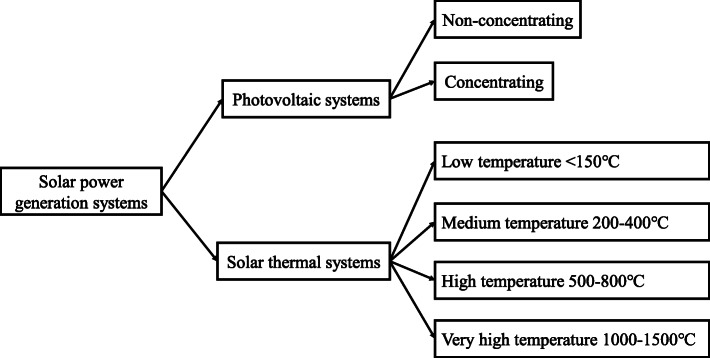
Main applications of using solar energy [54]

Solar energy is a promising and freely available renewable energy source for the energy transition. The PV and solar thermal are two kernel technologies for using solar energy as shown in Fig. 16 [50–52]. Solar thermal works by using mirrors to concentrate sunlight first. Then, the concentrated solar energy is transformed into stored heat to generate electricity [51]. The solar PV directly converts the sunlight into electricity using semi-conductors [52]. As the main and the most important usage of solar energy, solar PV technology has been developed rapidly in the past 20 years and this brought about a rapid reduction in the cost with price parity achieved on the grid. The solar PV power generation can be further divided into distributed and centralized power stations according to the scale of PV panel arrays. For the distributed style, the PV panels are usually located on rooftops, in rural and commercial areas. The centralized PV power stations usually occupy a large area of land to form a certain scale. In China, the centralized solar PV power plants now account for about 69% of the total installed PV capacity by June, 2020 [53].

In 2020, China increased 48.2 GW of solar power capacity, which is far ahead of any other country. Now China has the world’s largest cumulative installed solar power capacity, with 253 GW at the end-2020 compared with about 151 GW in the European Union and 93 GW in the United States, according to International Energy Agency data [55]. Thus, solar PV power generation is a key technological and industrial support for realizing the carbon neutrality commitment in China.

**Fig. 17:**
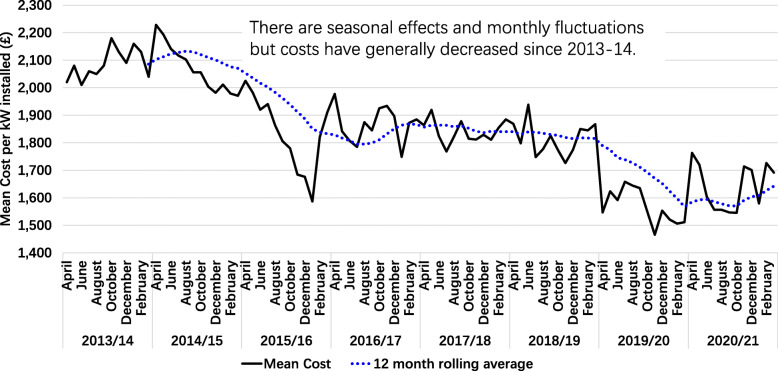
Mean cost per kW of 0-4 kW solar installations. (generated by the authors, data is from [56])

Driven by the target of carbon neutrality, the proportion of solar energy in China’s total energy will increase from about 2.7% to more than 25%. Considering that solar PV power generation involves many upstream and downstream industrial chains, this will provide a large number of jobs and open up a huge development space for the solar energy industry. In the past decade, the costs of solar PV have generally decreased since 2013 despite the seasonal effects and monthly fluctuations [56], as shown in Fig. 17. Although prices have rebounded this year due to the rising prices of bulk products and raw materials, there is still room for a further decline in the cost of PV power generation with the progress of solar PV power generation technology and the exponential development of the solar PV industry.

China is playing a leading role in the solar PV industry chain of the world, including the manufacturing capacity, completeness of the industrial chain, industrialization technology, manufacturing cost, and market scale. However, there is still great potential for China to make further progress in fundamental research related to solar energy. With the promotion and application of advanced solar technologies, China still needs to strengthen the capabilities of research and development, standards-setting, proficiency testing and verification. In the future, improving the utilization level of solar PV power generation is still the key to further increasing the proportion of solar energy consumption. This involves a series of issues including alleviating the impact of renewable energy on the grid, grid resilience, energy storage technology, etc.

#### Wind energy

**Fig. 18:**
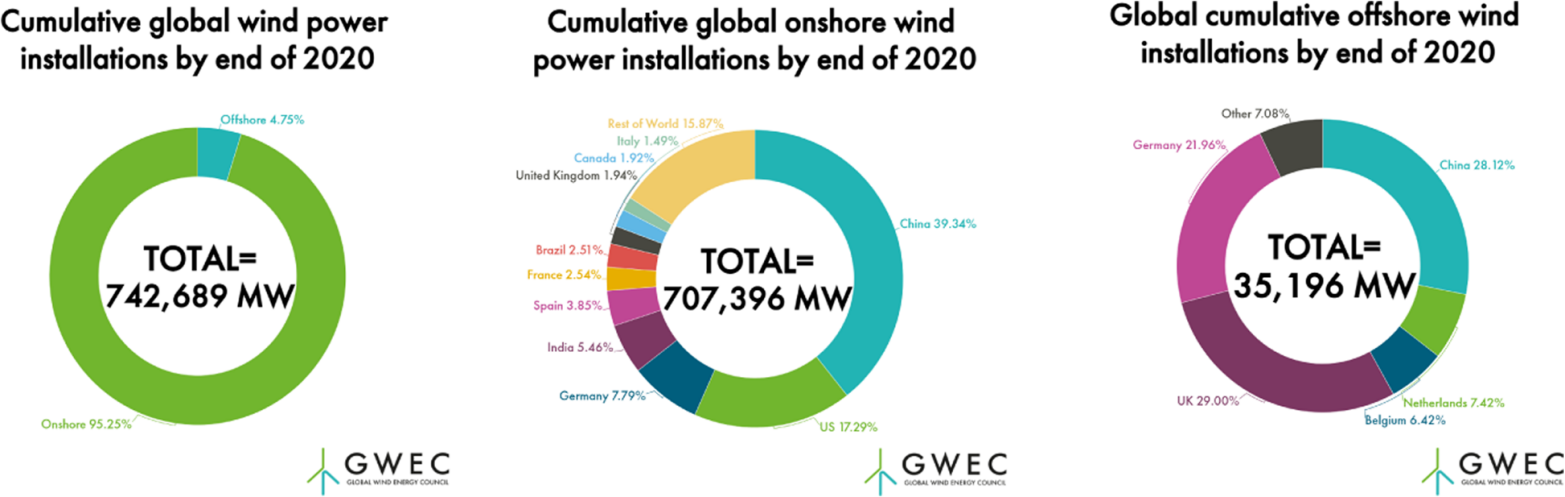
Cumulative global onshore and offshore wind power installations by end of 2020. Figure is adapted from [58]

Similar to solar energy, wind energy is another sustainable energy source for the energy transition in China [57]. According to the data released by the Nation Energy Administration (http://www.nea.gov.cn/), in 2020, China added 71.6 GW of wind power generation capacity to reach a total capacity of 281GW which is around 38% of the world’s total installed wind power capacity. Both China’s installed capacity and new capacity in 2020 are the largest in the world by a wide margin compared with the next largest market, the United States, adding 14 GW in 2020 and having an installed capacity of 118 GW. The installed capacity of onshore wind power in China reaches 278 GW by the end of 2020, which occupies 39.34% of the global onshore capacity and is more than two times of the United States as shown in Fig. 18. By the end of June 2021, China had increased its utility-scale offshore wind electricity generation capacity to 11.13 GW, rivaling the approximately 10.4 GW of installed capacity of the UK at the end of 2020, according to the data from China’s National Statistics Administration. By the end of 2030, China is expected to reach a cumulative grid-connected wind capacity of 689 GW, accounting for 67% of the global share.

Wind power generation integrates multiple technologies, including material research and development, blade design, hub/bearing/generator manufacturing, etc. The exponential growth of wind power generation will promote the rapid development of the wind power industry chain. Similar to solar PV power generation, the wind energy companies in China have formed a complete and internationally competitive industrial chain and occupy half of the global wind power industry. This is another guarantee for China to achieve the final carbon neutrality goal. Due to land constraints, the development of onshore wind power in China has gradually slowed down. Considering that China has a coastline of 18,000 kilometers, offshore wind power generation will increase a lot in the near future.

In 2020, the total installed capacity of solar PV and wind power generation in China reaches 533 GW (Table 4). Stimulated by China’s target of 1200 GW of wind and solar set for 2030, 408 GW of new capacity will be added from 2021 to 2030. In order to ensure the achievement of the carbon neutrality goal in 2060, China needs to increase the installed capacity of wind and solar power generation at least to 5000 GW by the end of 2050. Therefore, it is estimated that the wind and solar energy industries will keep developing rapidly in the next 30 years.

**Table 4:** Comparison of new installed and cumulative capacity of solar and wind energy since 2016. Data is from the National Energy Administration (http://www.nea.gov.cn/)

Year	New installed capacity (GW)		Cumulative capacity (GW)	
	wind	solar	wind	solar
2016	19.30	34.54	149	77.42
2017	15.03	53.06	164	130
2018	20.59	44.26	184	174
2019	25.74	30.11	210	204
2020	71.67	48.20	280	253

#### Energy storage

Renewable energy technologies such as wind and solar always face the problems of volatility, intermittentness, and uncertainty during energy generation. Simply developing renewable energy technologies will inevitably bring about problems such as the vibrational impact on the power grid and a high rate of abandoned wind and solar energy. The energy storage technology can effectively alleviate the above-mentioned problems by storing and releasing the intermittent and fluctuating energy [57, 59]. The Chinese government has paid great attention to the development of energy storage technology and industry. The National Development and Reform Commission and the National Energy Administration in China have successively released documents and guidance on promoting the development of energy storage [60, 61]. According to the national plan, the scale of new installed energy storage capacity (excluding pumped storage) in China will increase from the current 3–4 GW to more than 30 GW in 2025, making China the world’s largest energy storage market. By the end of 2050, the global energy storage capacity in the world is expected to reach 1600 GW (5500 GWh), and the cumulative installed energy storage capacity will exceed 200 GW (700 GWh). With the rapid growth of renewable energy power generation, the energy storage industry will also leap forward at the same time. The combination of renewable energy and energy storage will become important components of future energy systems.

**Fig. 19:**
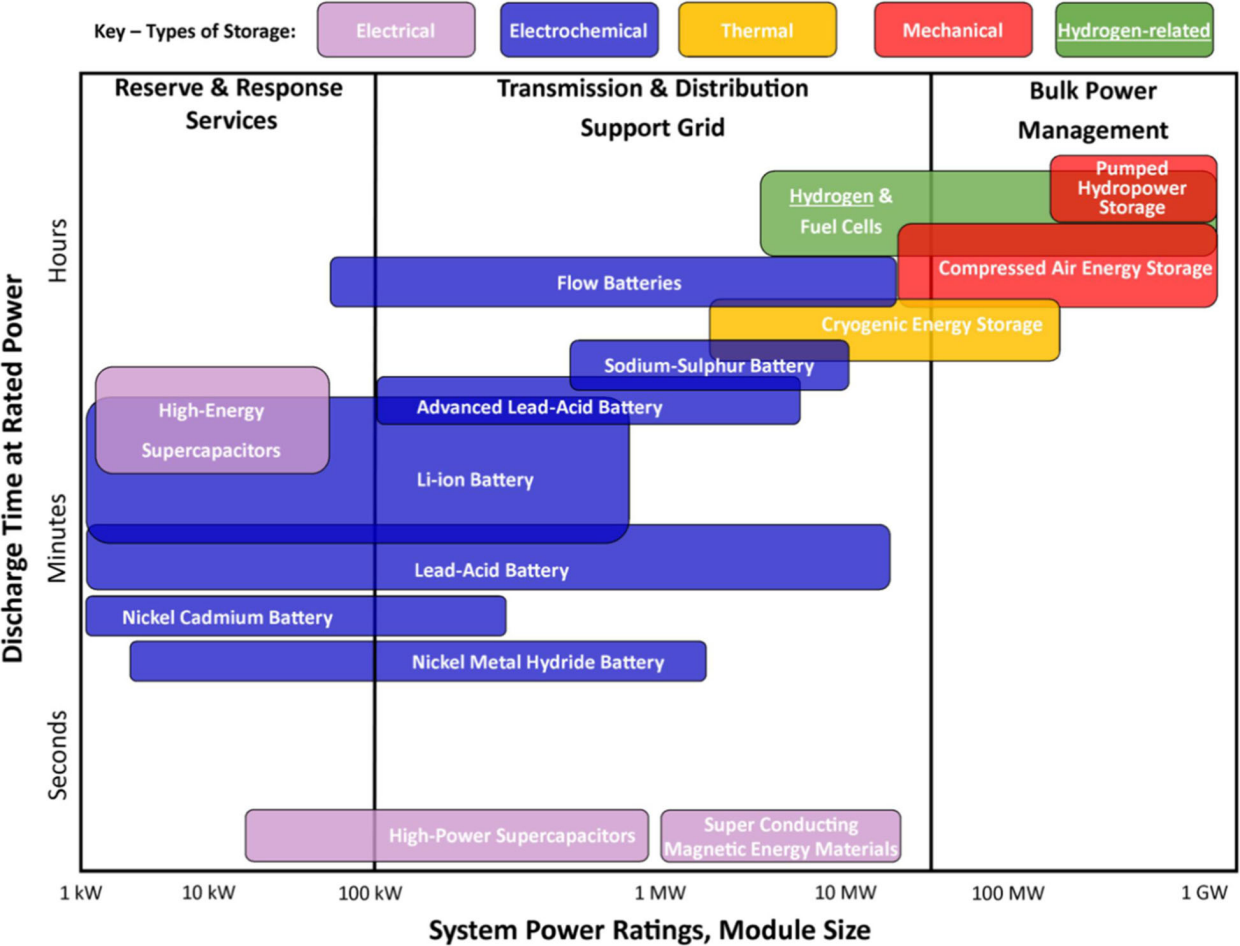
Comparison of various types of energy storage technologies [63]

Energy storage is the charging and discharging of energy through a reversible process, including mechanical energy storage, electrochemical energy storage, electromagnetic energy storage, thermal energy storage, etc. [62–64] The broad concept of energy storage also includes the conversion of renewable energy to chemical energy for storage, including hydrogen energy (hydrogen production by electrolysis of water), synthetic fuel (reverse conversion of carbon dioxide into fuel), biomass energy, etc. The evaluation of energy storage technology usually includes the rated power, rated capacity, response time, charging/discharging efficiency and stability. Different energy storage technologies have their own advantages, disadvantages and application scenarios (Fig. 19). The main driving force for the development of energy storage technology in recent decades has been the large-scale application of electric vehicles, mobile phones and laptops. In the future, the high demand for stable, continuous and reliable new energy power generation will become another important driving factor for the development of large-scale energy storage. Among different energy storage technologies, the dominant energy storage technology is the electrochemical energy storage. On one hand, solar and wind energy will become the main energy supply in the near future. On the other hand, the terminal utilization of human energy in the future will be electric energy. All of those determine that future energy storage will be mainly based on electricity storage.

The advanced electrochemical energy storage includes lithium-ion batteries, sodium-ion batteries, flow batteries, etc. Lithium-ion batteries are widely used in mobile phones, laptops and electric vehicles due to the advantages of high energy density, rapid response, and high cycle times. Meanwhile, lithium-ion batteries can catch fire or explode if they have been improperly manufactured or damaged, or overheated. With the development of thermal management technology, this serious problem has also been alleviated to a certain degree.

Currently, more than 70% of the lithium supply in China depends on imports [65]. Therefore, the development of alternative technologies such as sodium-ion batteries has also become an alternative way for China to solve the bottle-neck problem since the reserves of sodium are much richer. Compared with lithium, sodium-ion batteries have the advantages of low cost, no over-discharge, and high safety due to their chemical characteristics. Meanwhile, the energy density of sodium-ion batteries is correspondingly lower than that of lithium-ion batteries because the molecular weight of sodium is higher than that of lithium.

The flow battery has the advantages of large capacity, high cycle times, and large-scale storage of new energy since the electrolyte can be separated from the positive and negative electrodes. However, the energy density of flow batteries is lower than that of lithium batteries, and the cost is relatively high. The current manufacturing cost of lithium batteries is between 1,000 and 2,000 $\yen $/kWh, while flow batteries are more than 2,000 $\yen $/kWh. With large-scale manufacturing and application, the price of flow batteries is expected to keep decreasing.

With the motivation of carbon neutrality, the future electrochemical energy storage has a huge development space. Take the lithium battery as an example, the small battery involves various industries, including positive and negative materials, electrolytes, dispersants, and films. The massive demand for lithium batteries has driven the rapid development of entire industry chains. By the end of 2019, the new installed capacity of electrochemical energy storage in China reaches 0.64 GW, and the cumulative installed capacity has reached 1.71 GW. In 2020, the cumulative installed capacity exceeded 2 GW. By the end of Chin’s 14th Five-Year Plan, the capacity of electrochemical energy storage power stations will reach more than 20 GW. With the large-scale application of various energy storage technologies, the cost has also been significantly reduced. Taking lithium batteries as an example, the energy density and cycle life have been both doubled, and the application cost has been reduced by more than 70% during the past five years.

In addition to electrochemical energy storage, the thermal energy storage technology also has broad applications in district heating, mobile heating vehicles, distributed solar heating, combined cooling, heating and power systems, and solar water heating systems, etc. The temperature range for thermal energy storage application varies widely from −160 ^∘^C to 1000 ^∘^C [54]. Thermal energy storage technology can be divided into three main types: sensible heat storage, latent heat storage, and thermo-chemical energy storage. The energy storage density for the three types is gradually increasing, but the technological maturity is gradually decreasing. The sensible heat storage has been used in industrial applications, such as solar thermal power generation technology which uses molten salt as the heat storage medium. The medium and low temperature latent heat storage has been used in heating scenarios, while the medium and high temperature latent heat storage has not yet been commercialized on a large scale. Compared with sensible and latent heat storage, thermo-chemical energy storage is much more complicated and still at the laboratory research level.

#### Hydrogen energy and synthetic fuel

**Fig. 20:**
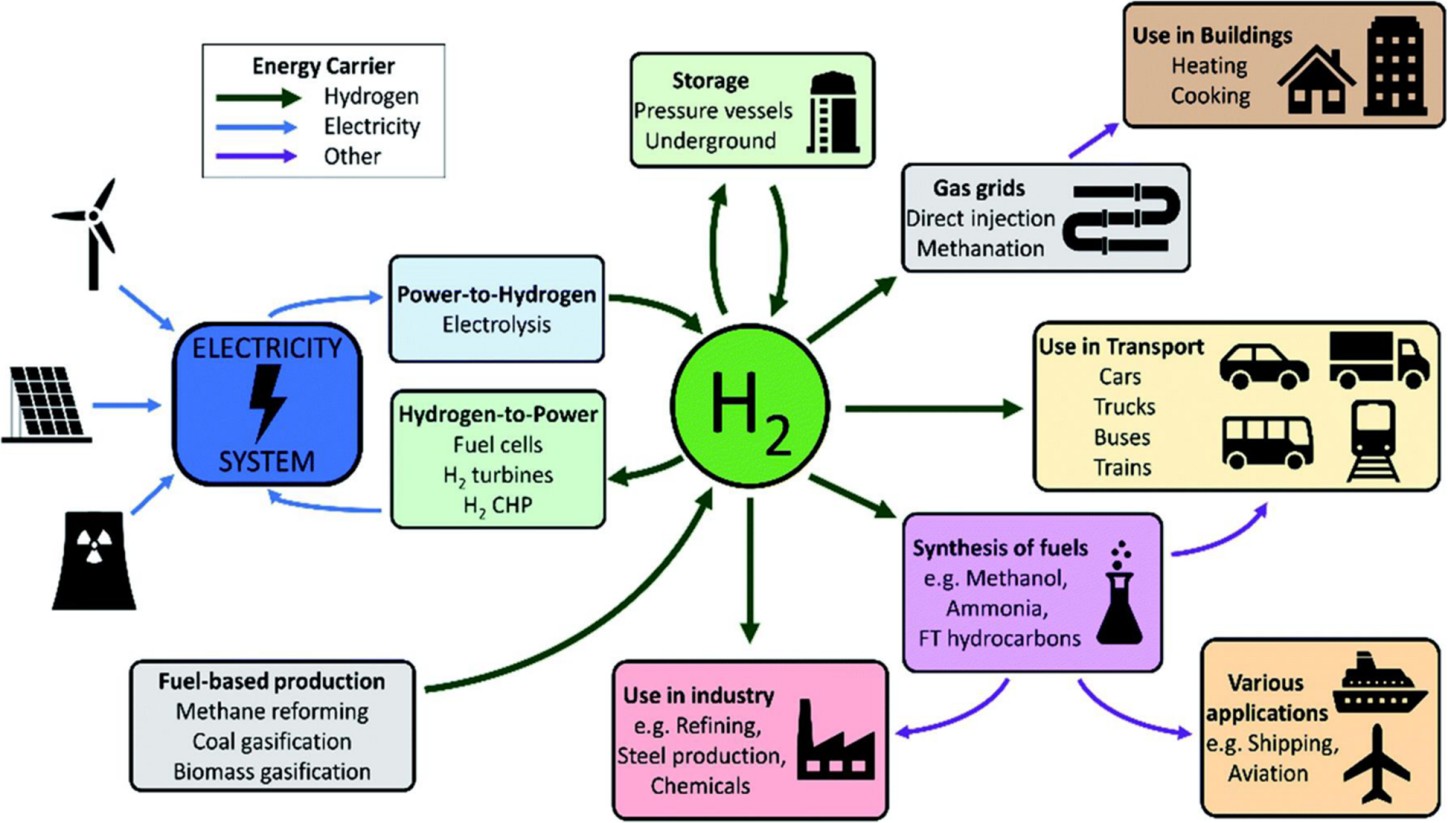
Overview of hydrogen production and usage pathways in energy systems [67]

Hydrogen energy is another important way of energy storage in a broad sense, and it is a clean, efficient, safe and sustainable secondary source of energy without CO_2_ emissions [66–68], and plays important role in energy systems as shown in Fig. 20. Hydrogen and fuels derived from hydrogen make up 6% of final energy consumption in China in 2060. Hydrogen can be used as fuel taking place of fossil fuels which is widely used in heavy industry, fuel-cell vehicles and making ammonia. If evaluated by unit mass, the calorific value of hydrogen is the highest among all fuels, which is three times that of oil and four times that of coal. As the product of hydrogen combustion is only water [69], the direct combustion of hydrogen to obtain energy is also an effective way to achieve a zero-carbon energy supply. According to different energy sources for hydrogen generation, hydrogen can be subdivided into gray hydrogen, blue hydrogen and green hydrogen. Gray hydrogen is produced through the combustion of fossil fuels, green hydrogen is produced by using renewable wind/solar energy to electrolyze water, and blue hydrogen generation is through the combination of fossil energy combustion and carbon capture technology to achieve carbon neutrality. The future path for hydrogen production is to produce hydrogen through the electrolysis of water using renewable energy [70, 71], which is based on the utilization of renewable energy and large-scale energy storage. This contributes a lot to decreasing CO_2_ emissions. The current hydrogen production in China is mainly based on coal, while it is by natural gas in other countries. At the current stage, the hydrogen production by electrolysis of water only accounts for about 4% of the total hydrogen supply.

Under high pressure, hydrogen molecules will pass through the metal walls, causing hydrogen leakage. Therefore, the storage of hydrogen needs high requirements. The current hydrogen storage is mainly divided into gas, liquid and solid style. High-pressure gaseous hydrogen storage and low-temperature liquid hydrogen storage have been widely used in the aerospace field. The organic liquid hydrogen storage and solid-state hydrogen storage technologies are still in the demonstration stage. Hydrogen can be stored and transported on a large scale, which is an important feature different from battery energy storage. The storage performance and transportation efficiency of hydrogen are current bottlenecks in the construction of hydrogen energy networks.

Hydrogen energy plays important role in fuel cells, which directly converts the chemical energy of hydrogen and oxygen into electrical energy. This technology has the advantages of no pollution, no noise, and high efficiency, but the high cost is still the main reason that limits its large-scale application. In the future, two problems still need to be solved for the large-scale hydrogen energy application. The first one is the cost of fuel cell stack manufacturing, and the other one is to improve the corresponding infrastructure constructions, including the hydrogen refueling stations, hydrogen pipelines, and hydrogen storage units.

Hydrogen production can be combined with the consumption of renewable energy to achieve large-scale storage of renewable electricity. Before the large-scale promotion of electrochemical energy storage, the production of hydrogen from renewable electricity will become an effective way to solve the problem of abandoning solar and wind. The future hydrogen production route will inevitably evolve from the current non-green or light green to the final dark green stage. The transition in the energy structure will inevitably bring evolutions in the production route of hydrogen. It is estimated that hydrogen energy will account for about 10% of China’s energy supply in 2050, and the demand for hydrogen will be around 60 million tons. Therefore, there is a large development space for producing hydrogen in the way of electrolysis of water using solar and wind energy.

In addition to using renewable electricity to produce hydrogen, the reverse synthesis of carbon dioxide to fuel has also become an important way for energy storage in a wide range. In 2017, Jaramillo et al. [72] reported the electrocatalytic reduction process to convert solar energy and wind energy into chemical energy for storage using nitrogen, carbon dioxide, water and other air components as raw materials, achieving controllable conversion and storage of clean energy. In 2018, Bai et al. [73] looked forward to the prospects and plans of using carbon dioxide as a raw material to convert intermittent solar energy into renewable liquid synthetic fuels. This technology route for storing wind and solar energy through renewable fuels has great potential applications. The European Union has announced the full use of synthetic fuels based on renewable energy by 2050. The one-way conversion process of fossil fuels to carbon dioxide has led to an imbalance in the distribution of carbon in the various layers of the earth. Through the reverse conversion process of carbon dioxide, including photocatalytic conversion, biochemical conversion, thermo-chemical conversion, electrocatalytic conversion, etc., not only energy storage can be realized, but also the purpose of carbon sequestration can be achieved, and finally a carbon-neutral cycle process can be realized.

The synthetic fuels using carbon dioxide also faces some technical challenges: poor selectivity for small molecule products, the low energy conversion efficiency of electrocatalytic carbon dioxide reduction, effective separation of liquid phase products, various ions transportation in a gas-liquid-solid three-phase environment, the thermal management issues, and the challenges of devices. Therefore, the synthetic fuels by electrocatalytic carbon dioxide reduction require the cross integration and collaborative innovation of multiple disciplines such as physical chemistry, energy materials, and engineering thermophysics.

#### Carbon capture, utilization and storage (CCUS)

The achievement of the carbon neutrality goal depends not only on the large-scale use of non-chemical energy, but also on the effective management of carbon dioxide emissions from steel, cement and chemical industries, as well as the combustion of fossil energy. The CCUS is currently believed to be an effective way to quickly neutralize carbon dioxide emissions [74]. However, the long-term impact of carbon dioxide storage on the ecological environment still needs to be further evaluated.

**Fig. 21:**
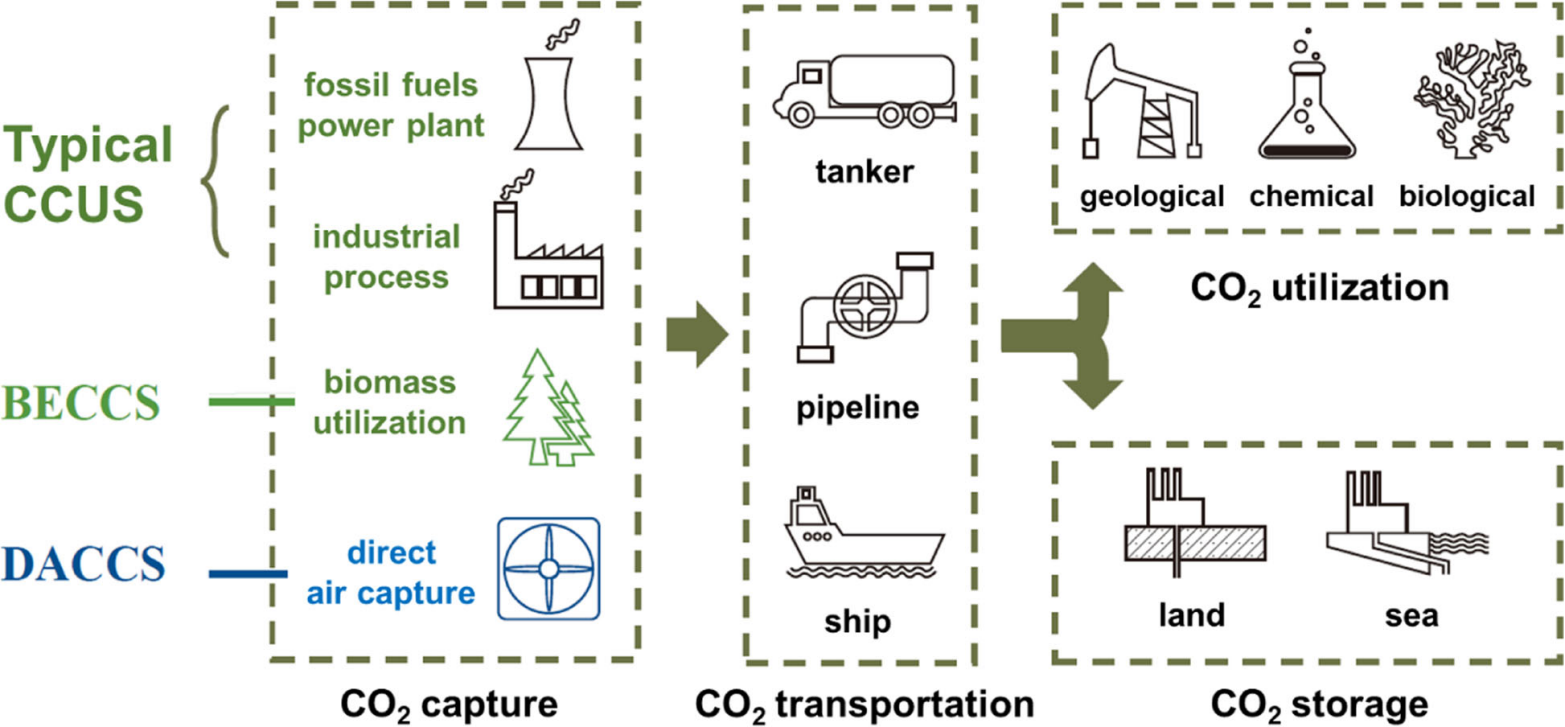
Technical processes of CCUS [75]

The key technical processes involved in CCUS are the CO_2_ capture, transportation, utilization, and storage, as shown in Fig. 21. The capture process is to separate CO_2_ from fossil fuel combustion, industrial production, or directly from air. The capture technology covers the pre-combustion, post-combustion, oxy-fuel combustion, and chemical looping combustion capture. The captured CO_2_ will be transported either by tanker, pipeline, or ship for further utilization, which mainly includes three categories: geological, chemical and biological utilization. The CO_2_ storage mainly stores liquefied carbon dioxide in abandoned coal seams or oil and gas fields, saline aquifers, and deep seabed. While achieving CO_2_ storage, injecting high-pressure liquid or supercritical CO_2_ into oil wells or natural gas fields can improve the oil and gas recovery rate. Besides the typical CCUS, the bio-energy with carbon capture and storage (BECCS) and direct air carbon capture and storage (DACCS) also become more and more attractive carbon dioxide removal technologies. The BECCS extracts bioenergy from biomass and captures and stores the CO_2_, thereby finally removing it from the atmosphere. The DACCS technology captures CO_2_ directly from ambient air and can be used with very low CO_2_ concentration.

**Fig. 22:**
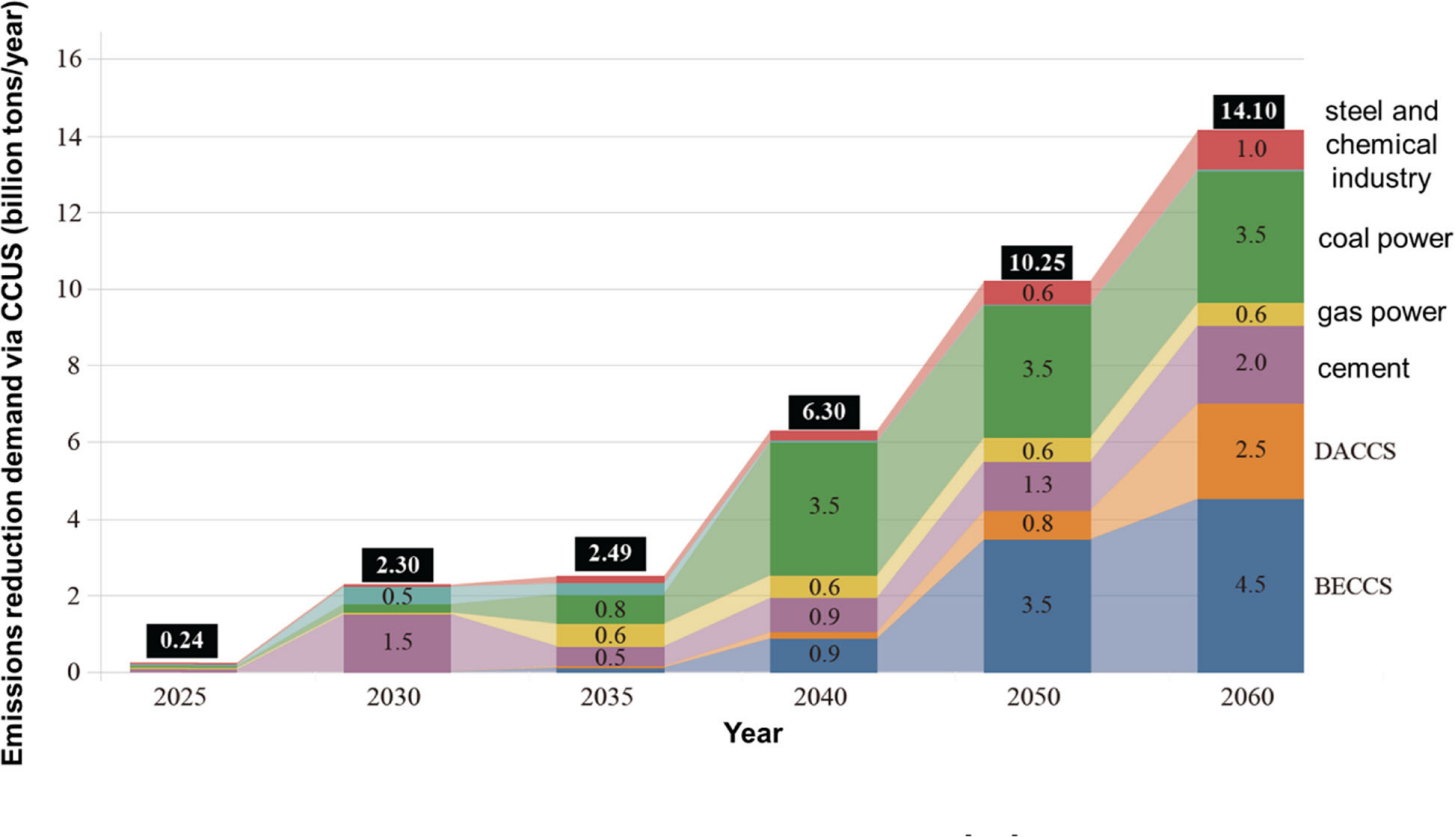
Emissions reduction demand via CCUS in China [75]

According to the annual report on CCUS in China 2021 [75], the emission reduction demand via CCUS in China under the carbon neutrality target is 20 to 408 million tons in 2030, 0.6 to 1.45 billion tons in 2050, and 1.0 to 1.82 billion tons in 2060, as shown in Fig. 22. China has promoted CCUS technology and launched over 40 demonstration projects with carbon capture capability of 3 million tons/year. Those projects were mainly conducted in the oil, coal, and electricity industry with small-scale capture capability, lucking of large-sale, combinatorial of multi-technologies, and whole-process demonstration. The high technical cost limits the large-scale application of CCUS technology, which includes the economic and environmental costs. The economic cost covers the whole operation process of CCUS, including the capture, transportation, storage, and utilization. It is estimated that by 2030, the cost of CO_2_ capture will be 90-390 $\yen $/ton, and 20-130 $\yen $/ton in 2060. The pipeline will be the main transportation method for large-scale CCUS demonstration projects in the future. It is estimated that pipeline transportation costs will be 0.7 $\yen $/(ton ·km) in 2030 and 0.4 $\yen $/(ton ·km) in 2060. For storage, the cost will be 40-50 $\yen $/ton in 2030 and 20-25 $\yen $/ton in 2060. The high technical cost and financing also mean that the promotion of CCUS technology requires further finical policy support from the government.

#### Energy internet and smart energy

**Fig. 23:**
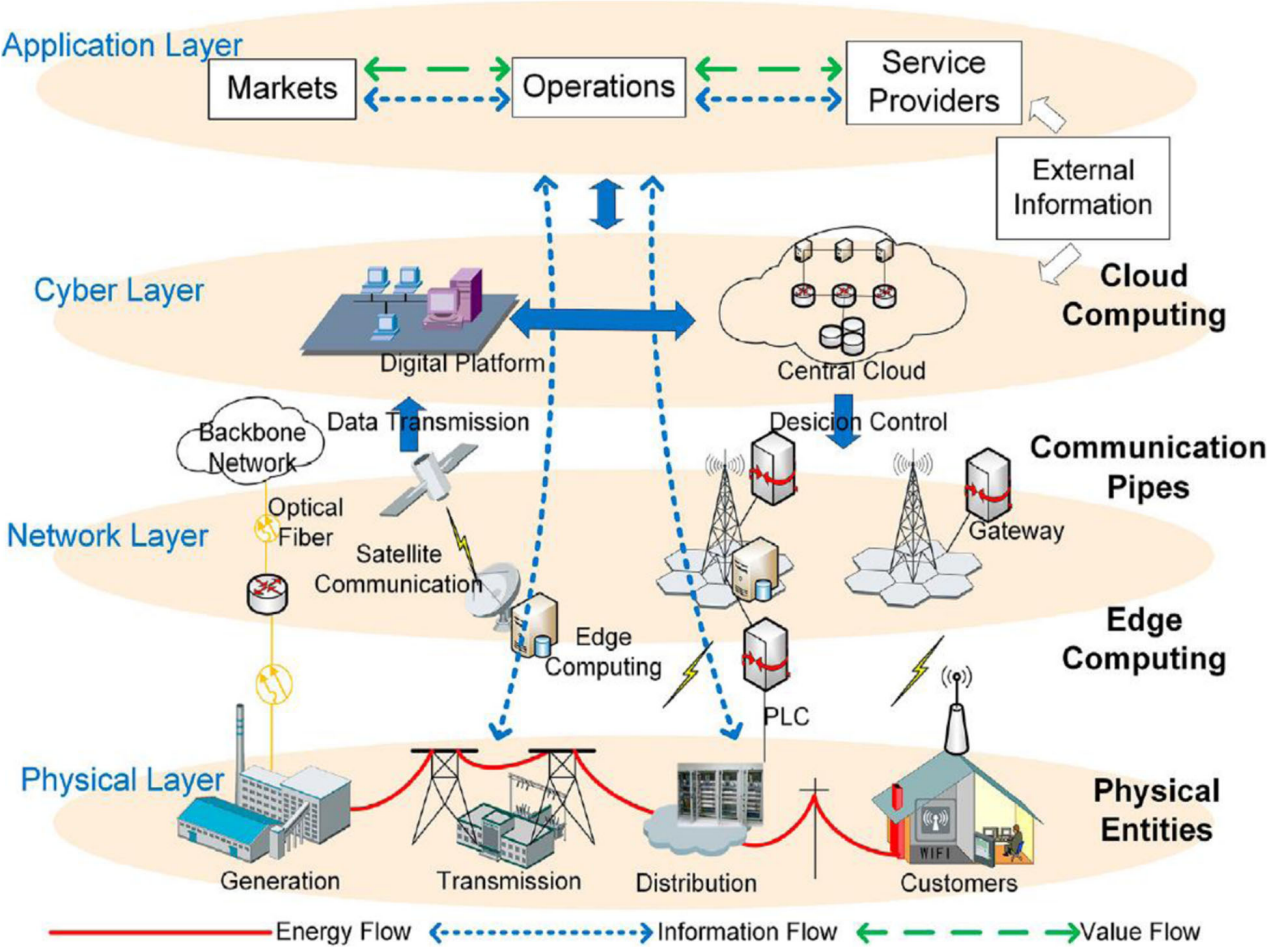
Architectural of the energy internet [78]

In the future, the deep integration of renewable energy and information technology will form the energy internet and smart energy system, which is a combination of distributed energy gathering devices, distributed energy storage devices and various types of loads, etc [76, 77]. The energy nodes are intelligently interconnected to achieve an energy reciprocal exchange and sharing network with a two-way flow of energy. Thus, the basic characteristics of the energy internet are renewable, distributed, interconnected, intelligent, open, and commercialized. From the architectural point of view, the energy internet can be generally divided into three levels as shown in Fig. 23, including the physical layer, the network/information layer, and the business/application layer. The key technologies involved in the energy internet include novel power generation technology, novel power transmission technology, power distribution technology, advanced energy storage technology and information and data technology.

With electricity as the mainstay, the distributed energy system involves multiple energy complements such as water grids, optical grids, and gas grids will be the development trend of future energy supply models. The distributed energy system uses wind and solar energy for the electrolysis of water to produce hydrogen, and it also combines biomass energy and municipal solid waste power generation technology. This system can intelligently dispatch, manage and recycle the supply of various forms of energy according to user-side demands, and finally achieves a high efficiency of energy conversion and usage.

The micro-grid is also an important part of the smart energy system. Compared with the distributed energy system, the micro-grid effectively connects the energy production side and the energy consumption side through big data, artificial intelligence, and information technology. It is foreseeable that the micro-grid will be a major way for human society to use energy in the future. The characteristics of decentralization and on-site energy production and consumption make the micro-grid different from the current energy networks. Inter-regional micro-grid interconnection which couples the cold, heat, and electrical loads between multi-regional micro-grid systems, can not only realize the horizontal multi-energy complementation between multiple micro-grid systems, but also realize the vertical source network. The interconnection and optimization of micro-grid between multiple regions can effectively increase the comprehensive utilization rate of energy. The individual in the micro-grid can first connect to the regional distributed energy supply system, and then further connect to the hub energy system of the town. Through this energy network structure, it is possible to effectively realize the ultimate goal and philosophy of everyone as an energy producer and energy consumer. This is of great significance for building an efficient, stable and safe energy network. China has also launched many demonstrations and promotions of Energy Internet/Smart Energy System, including Chongming Energy Internet, Suzhou Industrial Park, and Lingang Energy Internet Project [79].

We summarized the limitation and trends for the above-mentioned key technologies in Table 5. Generally, the high cost is one of the current main limitations for energy storage, hydrogen energy, synthetic fuel, and CCUS. The highly integrated CCUS and energy internet also depends on the construction of the infrastructure. This problem can be solved with the development of the industrial chain and as well as the support of carbon finance. Another typical trend is that those low carbon key technologies will develop mutually and promote each other, for example, solar/wind energy + energy storage, renewables + hydrogen energy, CCUS + synthetic fuel, etc. All those key technologies will contribute to the energy transition together.

**Table 5:** Comparison of the limitations and trends for different key technologies

Technologies	Limitations	Trends
Solar/Wind energy	volatility, intermittentness, uncertainty	cost reduction, integration with energy storage
Energy storage	high cost, safety, capacity	cost reduction, high capacity, long life, high safety, integration with renewable energy
Hydrogen energy	cost, safety (inflammable and explosive), transportation	green hydrogen by renewables
Synthetic fuel	cost, catalytic efficiency	integration with CCUS
CCUS	high cost of capture, transportation, storage and utilization, depend on infrastructures construction, luck of large-scale application	to be promoted by carbon trade, need finical policy support
Energy internet and smart energy	requires highly integration of hybrid energy infrastructures, network, information and business	renewable, distributed, interconnected, intelligent, open, and commercialized

## Climate investment and financing

In-depth transitions of China’s energy system are not an easy task and technology alone cannot do it, which requires extensive financial support. China has made tremendous efforts in the past years to increase government investment in climate change mitigation, adaptation and technological upgrade, implement tax policies to support green and low-carbon development, as well as strengthen innovation in climate investment and financing policies. According to the forecast of investment by multiple institutions, China’s carbon-neutral path means that by 2060, a total of 100–300 trillion Chinese Yuan of clean technology infrastructure investment opportunities will be generated. It is impossible to solely rely on the government for such a large funding. Therefore, how to actively utilize the resource allocation function of climate investment and financing through the design of policies, systems and mechanisms, and fully mobilize public and social capital is particularly important.

Relevant national ministries and commissions are also actively improving the top-level design, guiding and leveraging more social funds to enter the field to battle climate change. The national carbon trading market recently launched in Shanghai is an important channel for climate investment and financing. A healthy carbon market can stimulate the financial system to make a systematic response to the threat of climate change. The national carbon trading market initially only covered the power sector, but will ultimately cover other major emission sectors. Entities with annual emissions of 26,000 tons of CO_2_ in any year over the period 2013-2019 are required to participate in the first phase and 2,162 power plants meet these criteria, which covers total carbon emissions of about 4.5 Gt, accounting for 45% of the country’s current carbon emissions, is the largest carbon market worldwide. With such an ambitious scale of carbon market and related investment, studies are already showing that China will benefit greatly from driving the global low-carbon transition in the next 30 years. Carbon tax or other environmental taxes is believed to be another effective way of controlling GHG emission. However, before full implementations, its short and long term real effects on both economy and carbon reduction in China still require deep investigations [80].

### Carbon trade

Carbon trade theoretically is the most cost-effective policy that can equalize the marginal cost of emission reduction among the emitters in the market, and suppress the total emission below the targeting limit at least cost. Polluters with a higher cost of emission reduction are allowed to buy emissions from polluters that can abate at a lower cost. Benefits from carbon trading can be further used in energy-saving, emission reduction and innovations. To ensure the proper functioning of the emission trading system, effective allowance level, allocation rule, sector coverage and monitoring, reporting and verification (MRV) should be carefully considered and reasonably designed. Currently, more than 60 national and subnational jurisdictions are covered by emission trading scheme (ETS) initiatives.

As frequently criticized, the allowance price signaled by the emission trading system is highly volatile, which inhibits firms’ incentives to invest in emission reductions [81]. From this perspective, carbon price intervention performed by different policy instruments is investigated and introduced in practice. With price ceiling, price floor or both, the carbon price, therefore, fluctuates in the reasonable range. For example, Canada-Québec Cap-and-Trade System sets the minimum prices for auctioned allowance and increases 5% annual considering inflation [82]. Another price stabilization method is the market stability reserve (MSR), which is adopted by EU ETS at its fourth phase [83]. Additionally, carbon price and allowance level must be coordinated internationally to avoid carbon leakage from a carbon-constrained region to a non- or less-constrained one [84]. Possible measures to reduce carbon leakage are proposed by Zhou et al. [85] as real-time supporting policies (such as border tax adjustment) decided by market participation and limited quotas at the initial stage of emission trading.

An effective design of emission trading system should also understand the responses from producers and consumers at the micro-level, as the responses could be highly heterogeneous in terms of marginal abatement cost [86] or associated utility changes [87]. For example, the labor force in energy-intensive and high-polluting industries would suffer from higher adjustment costs. Thus, understanding responses from various players at different levels would be helpful to policy design with different regressive distributional effects.

A high level of carbon prices would be a great incentive for innovation in emission reduction. However, the overall cost of emission reduction would gradually decrease with the development of technological innovation, consequently resulting in the decline of the carbon price. Thus, the long-term stimulus of the carbon price to technological innovation could be highly limited due to the diminishing marginal utility. Lillistam et al. [88] examine the effects of carbon pricing on low-carbon investment, zero-carbon investment and innovation. No strong effect was proved by reviewing the ex-posts experience. Thus, complementary measures for ETS, such as innovation funds, subsidy policy, and financial support for fundamental research in renewable energy and emission reduction, should be taken into consideration.

Originally launched in seven provinces and municipalities, China’s carbon trading system/market further expanded nationwide on July 16^*th*^ 2021, after ten years of planning and trials. Currently, China’s ETS is designed to reduce CO_2_ intensity of economic activity rather than the total emissions [89]. During the first phase of China’s ETS, around 2200 companies in the power sectors, including combined heat and power, and on-site generators, are covered in the system. A total of 4.5 billion tone CO_2_ is regulated annually under the cap-and-trade system. As mentioned by Xiliang Zhang from Tsinghua University, the scheme will finally cover around 7500 enterprises and regulate 6.7 billion tone CO_2_. These companies are mostly large firms in eight typical energy-intensive sectors: electricity, buildings, iron and steel, non-ferrous metal processing, petrorefining, chemicals, pulping and aviation. Generally, the national allowance in China’s ETS is set according to national GHG emissions requirements, economic growth, economic structure, energy structure and coordinated control of air pollutants. At the enterprise level, the allocation plan consists of two parts: a pre-allocation of permits based on historical CO_2_ emission intensity and output and ex-post adjustments determined by actual output. Although the allowance is allocated without charge at present, it will gradually shift to paid form over time, according to the Administrative Measure and the Interim Regulation. Besides, the covered entities are allowed to use offsets for up to 5% of their verified emissions from CCER projects in renewable energy, carbon sinks, methane utilization and others.

### Carbon tax

Generally, carbon tax established a direct link between GHGs emission of a product or process and the corresponding tax needed to be paid. Similar to what ETS does, carbon tax puts a price on carbon, providing incentives for enterprises to mitigate emissions. However, significant differences exist between the carbon tax and ETS. As mentioned before, ETS regulates carbon emission by setting a maximum emission allowance. The carbon price formed by ETS is theoretically the lowest mitigation cost, but highly sensitive to climate and energy policies. On the other hand, carbon taxes provide a more stable signal to investors and are potentially a better choice where governments intend to provide multiple mitigation incentives. Currently, carbon taxation is implemented in more than 30 countries worldwide, most are European and American countries. And the price varies dramatically, ranging from $0.079 (Poland) to $137.24 (Sweden).

**Fig. 24:**
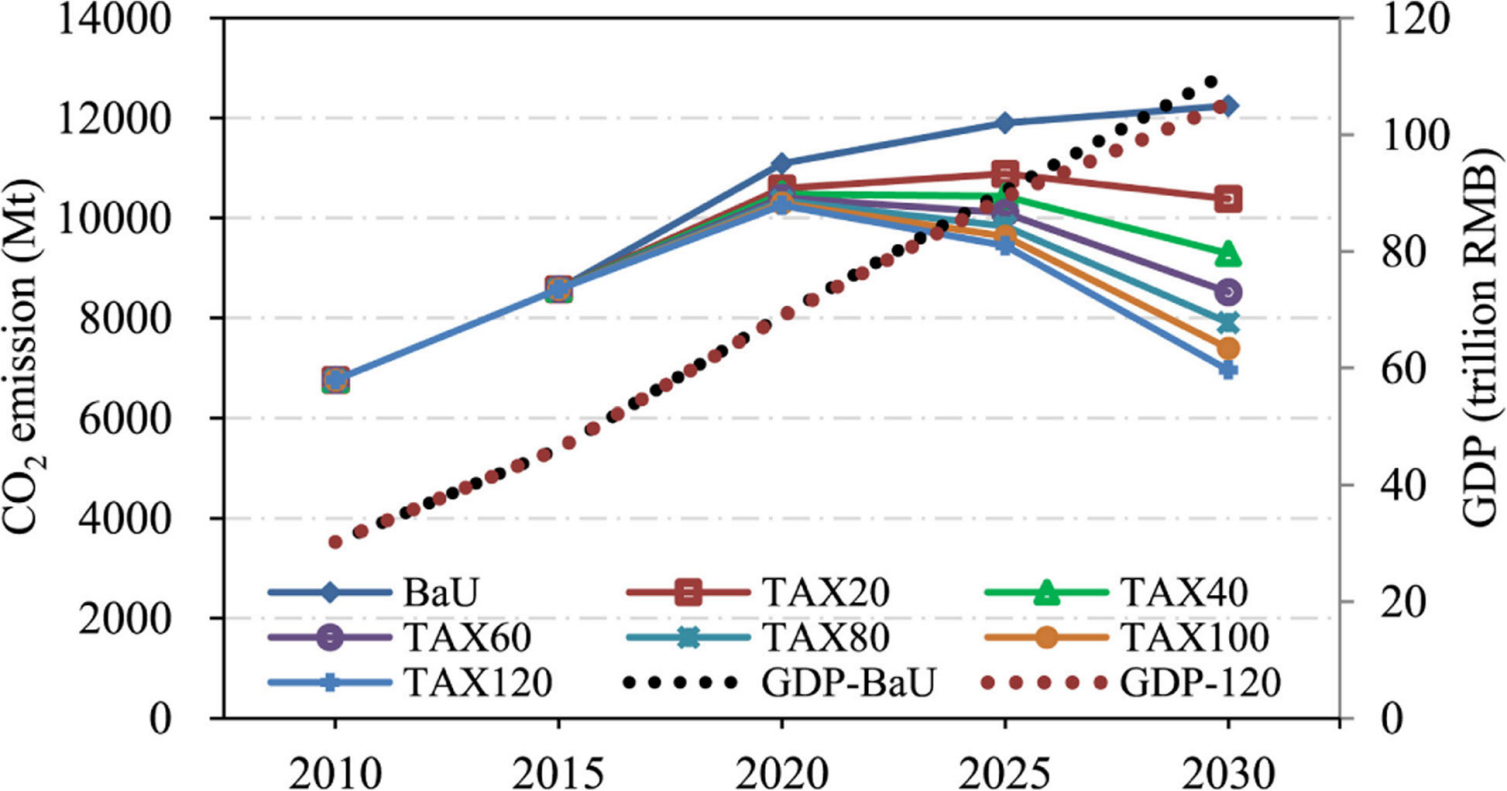
Effects of carbon tax on China’s industrial CO_2_ emissions [90]

Although not currently adopted in China, carbon tax is still advocated as one effective complementary measure for accomplishing targets of carbon peak and carbon neutrality. As shown in Fig. 24, Dong et al. [90] used a computational general equilibrium (CGE) model to evaluate the effect of carbon tax on 31 provinces. With the increase of taxation level from $0 to 120 /ton, the national CO_2_ emission reduced from 12.2 Gt to 7.0 Gt correspondingly. Carbon tax policy should be preferably implemented in electricity, metal melting and chemicals sectors to ensure the best utility of taxation. Besides, the carbon tax will result in GDP loss for all provinces, thus the carbon price should be no more than $50/ton. Aiming at minimizing the negative effects of welfare loss, the revenue from carbon tax should be reallocated and transferred more to western regions.

Lin and Jia [91] also conducted a CGE based study in China. Their results showed that the negative impact of carbon tax on GDP is limited to 0.5%. And the inclusion of both energy-intensive enterprises and energy industries under high tax rates would maximize emission reduction and maintain an acceptable impact of carbon tax on China’s GDP. Ding et al. [92] used a diffusion model of energy technology to explore the effects of different carbon tax conditions on carbon peak and technology evolution. Similar to the aforementioned research, a higher taxation level leads to a lower carbon peak. Transitional and advanced energy technology would appear much earlier with high carbon tax conditions than low ones.

In 2021, both ETS and carbon tax initiatives would cover 8.73 and 2.99 Gt CO_2_ equivalent respectively.

### Opportunity

**Fig. 25:**
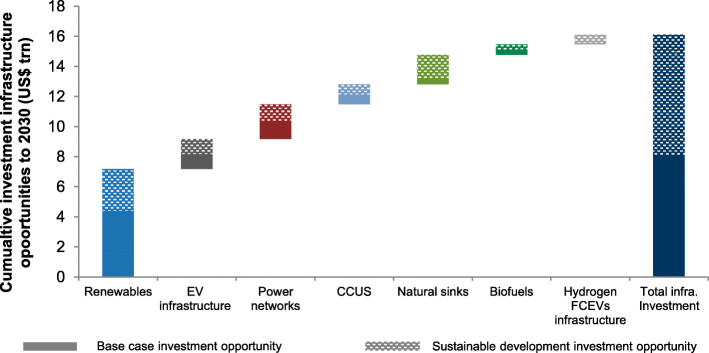
Cumulative global investment in clean energy transition to 2030 [93]

**Fig. 26:**
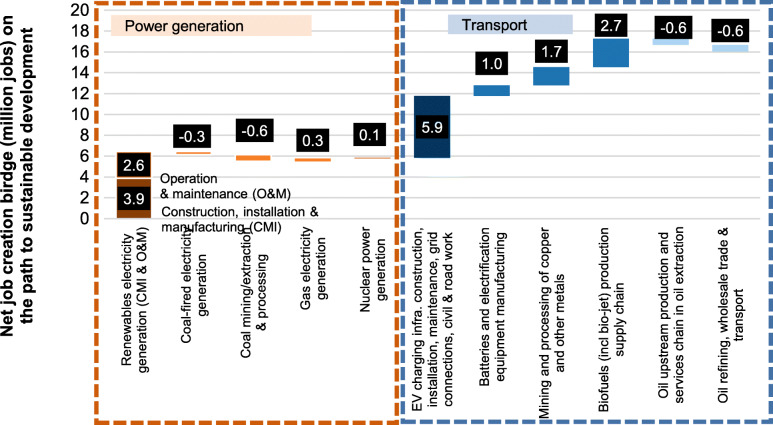
Global net job creation (mn) bridge for a sustainable path across the energy supply chain [93]

Through public-private collaboration, low financing costs and a supportive regulatory framework, climate or green infrastructure investments would be the green engine of the global recovery from COVID 19. According to a recent report by Goldman Sachs [37, 93], green infrastructures, characterized by solar PV, offshore wind, are 1.5-3.0 more capital- and labor-intensive than traditional energy development. Thus, as shown in Figs. 25 and 26, the development of clean technology would potentially drive a total investment opportunity of up to $16 trillion and bring 15-20 million jobs worldwide by 2030.

Over the last decade, the average cost of electricity from solar photovoltaic has fallen 82% and that of wind power has also dropped by one third. The fast-falling renewable energy prices lay the solid foundation for accomplishing carbon neutrality targets. Recent research published by He, Lin and Phadke et al. [94] indicates that if the falling trend of solar PV, wind, and battery storage continued, non-fossil sources would provide 62% of China’s electricity generation by 2030 at a cost 11% lower than business-as-usual condition, with a corresponding 50% emission reduction of the power sector from 2015 basis.

**Fig. 27:**
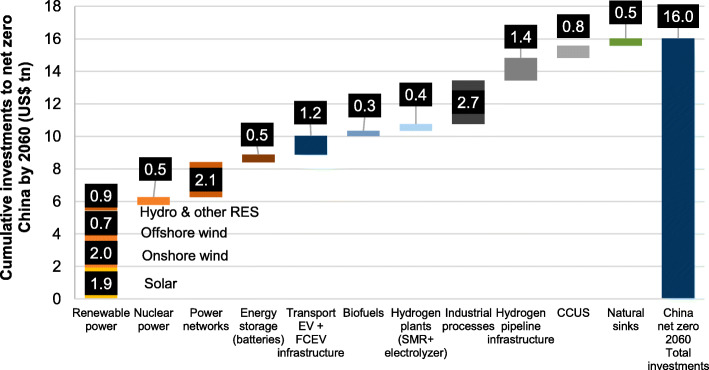
Cumulative investment opportunity across sectors for China net-zero by 2060 (US$ tn) [37]

**Fig. 28:**
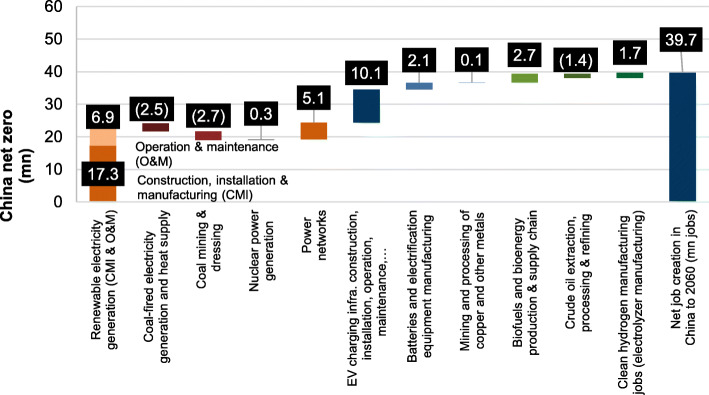
Net job creation bridge on the path to net-zero China by 2060 (mn) [37]

As predicted by Goldman Sachs [37], electrification through renewable power, clean hydrogen, carbon capture together will address more than 85% of China’s carbon emission by 2060. As the leading country of global carbon neutrality, China’s carbon neutrality path is estimated to provide $16-20 trillion green infrastructure opportunity and 40 million net new jobs by 2060 (Shown in Figs. 27 and 28). Sectors of New Energy Vehicles and renewable energy will be major beneficiaries in China’s low carbon development.

## Conclusion

China’s pledge to hit peak carbon emissions before 2030 and for carbon neutrality before 2060 is an important contribution to global efforts to tackle global warming and climate change. According to Climate Action Tracker, If achieved, China’s carbon neutrality would lower the projected rise in average global temperature by an estimated 0.2 to 0.3 ^∘^C [95]. However, China is now the world’s largest consumer of energy, the largest emitter of carbon dioxide and the primary consumption of fossil fuel energy is the leading source of more than 90% of China’s GHGs emissions. Reducing dependencies on high-carbon system and speeding up the deep transitions of the current energy structure is of paramount importance for China, and there are many challenges ahead. Achieving the carbon neutrality target is also helpful to address the energy security, safety and stability issues in China, and this will accelerate technological innovation and boost renewable capacity expansions. In this paper, we reviewed the carbon emissions, energy structures, and climate policies in China, analyzed the potential energy transition pathways to achieve the GHG emission reduction target with different technologies, summarized the energy transition related investment and financing opportunities. We believe that It requires practical and feasible policy guidance, technological breakthroughs and industrial upgrades, as well as adequate capital guarantees and carbon market mobilization to help China’s energy transitions toward carbon neutrality. On the path of the energy transition for carbon neutrality, we believe that decarbonization of fossil fuels, applying energy-saving technologies and CCUS is important in the early stage, followed by dramatically increasing the proportion of renewable and clean energy in the future energy supply, which needs to be supported by energy storage and smart energy system; the combination of renewable energy and energy storage technology will become more and more important. The energy internet and smart energy enable the combination of multiple renewable energy systems, the application of data and information technology for smart carbon emission management, will contribute to emission reduction.

## Data Availability

Not applicable.

## Abbreviations

APS: Announced pledges scenario
BECCS: Bio-energy with carbon capture and storage
CCUS: Carbon capture, utilization, and storage
CGE: Computational general equilibrium
COP: Climate Change Conference
DACCS: Direct air carbon capture and storage
EU-28: European Union plus the UK
FYP: Five Year Plan
GDP: Gross domestic product
GHGs: Greenhouse gases
Gt: Gigatonnes
HCFCs: Hydrochlorofluorocarbons
IEA: International Energy Agency
IPCC: Intergovernmental Panel on Climate Change
MEE: Ministry of Ecological Environment
MRV: Market stability reserve
MSR: Nationally determined contributions
NDC: Nationally Determined Contributions
NDRC: National Development and Reform Commission
NOAA: National Oceanic and Atmospheric Administration
PV: Photovoltaic
TOA: Top-of-atmosphere
